# Comprehensive biochemical, molecular and structural characterization of subtilisin with fibrinolytic potential in bioprocessing

**DOI:** 10.1186/s40643-025-00860-1

**Published:** 2025-03-21

**Authors:** Shreya S. Shettar, Zabin K. Bagewadi, Mohammed Alasmary, Basheerahmed Abdulaziz Mannasaheb, Ibrahim Ahmed Shaikh, Aejaz Abdullatif Khan

**Affiliations:** 1 Department of Biotechnology, KLE Technological University, Vidyanagar, Hubballi, 580031 Karnataka India; 2 Department of Medicine, College of Medicine, Najran University, 66462 Najran, Saudi Arabia; 3 Department of Pharmacy Practice, College of Pharmacy, AlMaarefa University, P.O. Box 71666, 11597 Riyadh, Saudi Arabia; 4 Department of Pharmacology, College of Pharmacy, Najran University, 66462 Najran, Saudi Arabia; 5 Department of General Science, Ibn Sina National College for Medical Studies, 21418 Jeddah, Saudi Arabia

**Keywords:** Subtilisin, Purification, Biochemical characterization, Applications, Molecular docking, Molecular dynamic simulation

## Abstract

Enzyme deployment is proliferating extensively in industries owing to their environmentally friendly and easily degradable attributes. This article undertakes an exhaustive examination of wild subtilisin enzyme, covering purification, biochemical delineation, analytical techniques, and practical implementations. The purification methodology involved partial refinement, anionic exchange, and gel filtration chromatography, culminating in a purification factor of 3.406, corroborated by SDS-PAGE showcasing a molecular weight of ~ 42 kDa. Biochemical scrutiny unveiled the enzyme's response, with an optimal pH at 9 and temperature peak at 60 ℃. Various surfactants, metal ions, organic solvents and inhibitors exhibited notable efficacy. Substrate specificity and kinetics showcased the utmost specificity with N-Suc-F-A-A-F-*p*NA, registering K_m_ and V_max_ values of 0.731 ± 0.5 mM and 0.87 ± 9 × 10^3^ U/mg, respectively. Different bioanalytical techniquesproffered insights into structural and biophysical facets. Practical applications encompassed goat skin depilation, feather disintegration, blood clot dissolution, exemplifying the enzyme's multifaceted utility. To embark upon the elucidation of structure–function relationships, a three-dimensional model was devised through homology modelling, leveraging existing subtilisin structures (PDB: 3WHI). Molecular docking score of − 8.8 kcal/mol and dynamic simulations augmented the comprehension of molecular interactions with N-Suc-F-A-A-F-*p*NA. This research significantly contributes to unravelling the biochemical intricacies of wild subtilisin and underscores potential industrial and biomedical prowess. Subtilisin can be explored for its thrombolytic potential in several cardiovascular diseases. It may aid in the management of thrombosis by dissolving blood clots in conditions like deep pulmonary embolism, myocardial infarction, ischemic strokes, and in atherosclerosis by breaking down fibrin in arterial plaques, thus preventing heart attacks and strokes.

**Graphical Abstract:**

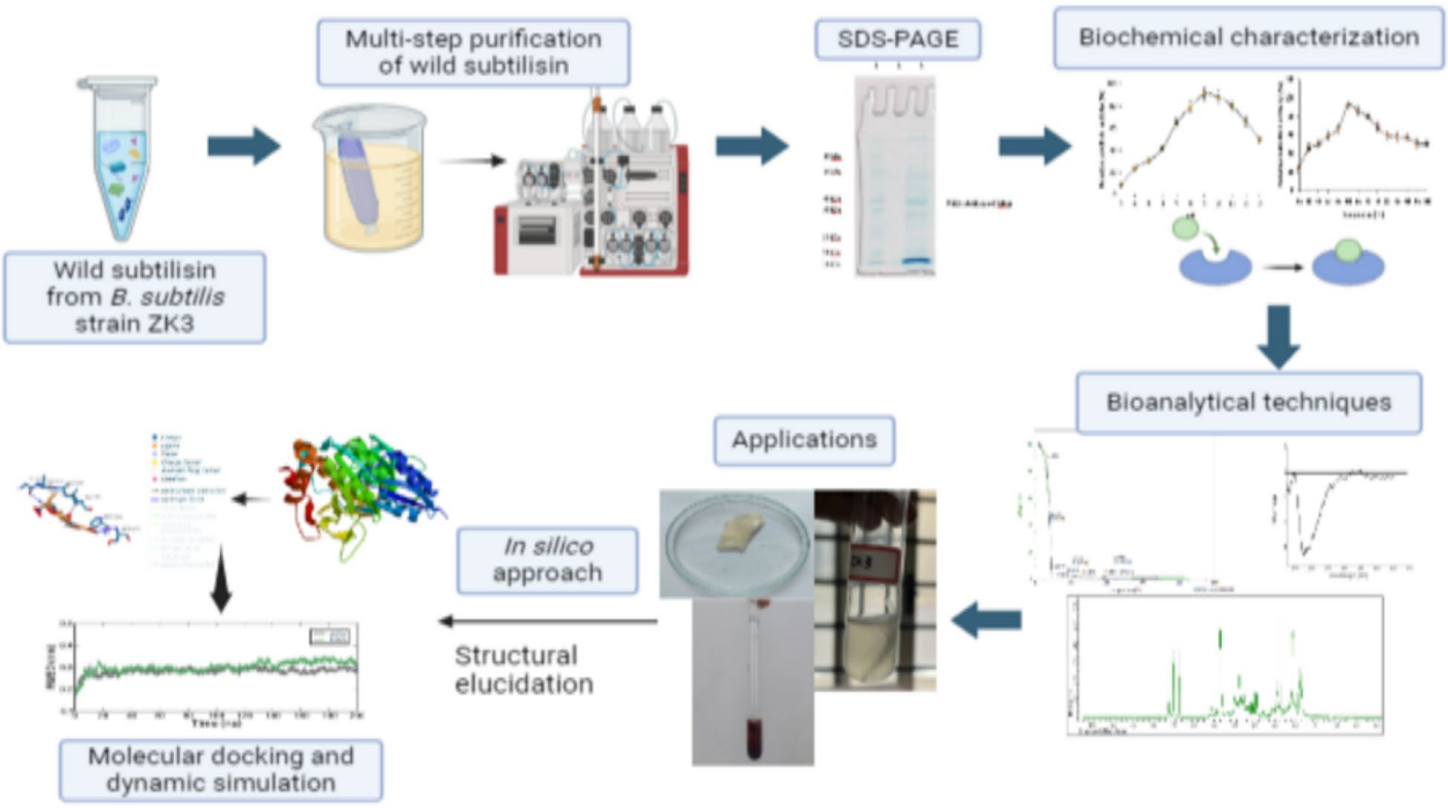

**Supplementary Information:**

The online version contains supplementary material available at 10.1186/s40643-025-00860-1.

## Article highlights

- A ~42 kDa subtilisin was chromatographically purified.
- Alkali thermostable subtilisin was molecularly characterized using CD, TGA and NMR.
- Structure-function relationships were established by homology modelling and molecular docking dynamic simulations.
- Subtilisin revealed diverse biotechnological applications.

**Supplementary Information:**

The online version contains supplementary material available at 10.1186/s40643-025-00860-1.

## Introduction

Every year, chicken processing companies throughout the world create countless pounds of plumage as a byproduct. This vast quantity of feathers serves as a significant waste output. In excess of 90% protein, feathers are crafted from keratin, an insoluble and fibrous structural protein. Feathers, characterized by their slow natural degradation, are classified as perilous waste. However, the decomposition aided by microbes with keratinolytic capabilities represents a second possibility to the biological transformation of keratin residues arising primarily from the poultry as well as leather sectors. Indeed, proteolytic keratin degradation has the potential to be used in case of hazardous chemical procedures and provides cost-effective and benign reaction circumstances enabling the synthesis of useful goods (Grazziotin et al. 2007; Fakhfakh-Zouari et al. 2010). Due to the increasing environmental pollution linked to the poultry industry, especially regarding keratin waste, the pursuit of efficient biocatalysts for keratin hydrolysis has emerged as a pressing goal for researchers (Bilal et al. 2024). Lately, alkaline proteases have garnered significant prominence in leather industries due to their capacity to safely and environmentally responsibly dehair animal skin. Lime and sulfide are utilized in the initial stage of conventional leather processing to remove hairs from animal skin (Sujitha et al. 2018). This method generates a significant amount of detrimental effluent, which has a negative influence on the soil as well as the water. Furthermore, a lack of rigorous monitoring of the chemical response might result in harm to the skin and the shedding of hair as well as yarn. Hence, proteases can function as a more environmentally friendly substitute, displacing the utilization of hazardous chemicals in the leather companies (Khandelwal et al. 2015; Matkawala et al. 2019). Maruthiah et al. (2016) found that APase from *Bacillus* sp. APCMST-RS7 possesses 80% maximal activity for scavenging DPPH radicals thereby acting as an antioxidant. Cardiovascular disease is one amongst the prime causes of illness and death globally. Proprotein convertase subtilisin/Kexin type 9 is a circulating protein that interacts with low-density lipoprotein cholesterol (LDL-C) receptors on liver cells, directing them toward lysosomal breakdown (Liu et al. 2021). The removal of biofilms has been shown to be highly effective using subtilisin-like protease and glutamyl endopeptidase. Enzymatic treatment resulted in the breakdown of EPS components and considerable disruption of biofilms (Mitrofanova et al. 2017). Another study advocates for the application of subtilisin as a biofertilizer and highlights the importance of recycling keratin-rich industrial waste for biofertilization purposes (Caballero et al. 2020).To overcome these environmental and cardiovascular issues, bio-products are used for effective utilization in industries and healthcareto reduce the hazardous treatments caused by chemical usage. Subtilases are among several multiple kinds of serine proteases transcribed in the genomes that comprise many dwelling forms, notably viruses. Subtilases are separated into six different groups according to the amino acids that constitute the sequence: subtilisins, thermitases, proteinases K, lantibiotic the peptidases kexins, and pyrolisins. Genuine subtilisins, extremely alkaline proteases, the intracellular proteases, in-between subtilisins, along with high-molecular-weight subtilisins are comprised of several the subsidiary families of subtilisins.Subtilisin A (EC 3.4.21.62), the initially discovered alkaline in nature serine protease that has been extensively executed, originates from *Bacillus subtilis*. The enzyme is named after the bacterial species that produces it (Ottesen and Svendsen 1970; Ikemura et al. 1987).The catalytic core of serine proteases are proteins composed of a trio unique amino acid such as: Aspartic aicd-32, Histidine-64, and Serine-221. Given that the amino acid component responsible for the attack that is nucleophilic is Serine-221, subtilisins and associated digestive enzyme family have been designated as serine proteinases. Proteases are predominantly manufactured using bacteria as microbial producers, and among them, the genus *Bacillus* is notably distinguished as a prime source. Enzymes like these serve their purpose in the manufacture of laundry detergents owing to their vast pH and temperature tolerance, which ensures outstanding efficiency and durability (Rozanov et al. 2021). (Liu et al. 2019) found that *Pseudoalteromonas* sp. H2's metalloAPase may metabolize collagen from pig and salmon skins, resulting in antioxidant peptides. Hydrolyzed collagen peptides with H2 exhibit antioxidant action, making them a suitable addition for the food and cosmetic industries. Fibrinolytic enzymes are proteolytic enzymes capable of dissolving clots in the blood via fragmenting down fibrin. In recent days, novel serine proteases have found to be acting as a potential anticoagulant (Sharma et al. 2019; Li et al. 2021). Serine proteases, such as subtilisin, have a widespread application in the leather products, fabric, food, and pharmaceutical sectors. Sixty percent of the worldwide industrial enzyme industry is represented by extracellular protease and amylase, showcasing a diverse array of uses in leather, detergents, food, and pharmaceutical sectors.

Proteases and subfamilies have been categorized as acidic, neutral and alkaline digestive enzymes depending on their respective pH measurements. Different types of serine proteases exhibit unique characteristics such as catalytic activity, substrate selectivity, temperature and pH preferences, stability profiles, and active site specificity (Naveed et al. 2021; Mohamed et al. 2022). In general, the thorough biochemical analysis of serine proteases is vital for both fundamental comprehension of enzyme function and pragmatic utilization across various sectors. This contributes to the advancement of effective and eco-friendly procedures in diverse industries. Comprehending the stability concerning pH and temperature facilitates fine-tuning the efficacy of serine proteases in industrial procedures like detergent production, leather processing, and pharmaceutical applications, evaluating the impacts of organic solvents guarantees the resilience of the enzyme in varied and demanding industrial settings, broadening its suitability and adaptability, examining the impact of metal ions is essential for recognizing possible cofactors, amplifying enzyme activity, or gaining insights into the inherent function of the enzyme. Assessing interactions with surfactants is crucial for customizing serine proteases for detergent applications, guaranteeing effective elimination of stains and contaminants. The proprotein convertase subtilisin/kexin (PCSK) 6 has been associated with many biological functions, involving embryonic development and tissue aging. Recent research involving human genetics and animal models suggests that PCSK6 functions in regulating cardiovascular function and disease progression (Wu and Chen 2022). Scrutinizing inhibitors assists in deciphering the enzyme's mechanism, offering perspectives for drug development and comprehending potential targets for therapeutic interventions. All these parameters are essential to study about the enzyme and its behavior (Vojnovic et al. 2024). The bioanalytical techniques complement each other by offering a comprehensive understanding of various aspects of serine proteases, including their stability, structure, conformational changes, and interactions. This multidimensional analysis is crucial for both fundamental research on enzyme behavior and the practical application of serine proteases in different industries. To reveal the molecular weight and atomic structure of serine proteases, techniques like Sodium Dodecyl Sulfate Polyacrylamide Gel Electrophoresis (SDS-PAGE), mass spectroscopy, X-ray Crystallography and Cryo-EM are used.

Due to their intricacy, experimental procedures are typically costly and executed at a low throughput. To address these challenges, *insilico* approaches have been established to discover prospective pharmacological targets. The advent of computer-based biological approaches has transformed life science research. Bioinformatics has significantly reduced the financial burden of laboratory experiments and minimized the need for animal testing in medical research. In silico techniques are valuable for classifying proteins by their structure and function. Additionally, methods like molecular docking facilitate the analysis of potential natural therapeutic ligands and receptor complexes. These tools also provide insights into previously unknown molecular structures, such as enzymes and their potential ligands, which hold great promise for future genetic engineering and biotechnological advancements (Sadeghi et al. 2021; Haghighi 2021). Target proteins are capable of being identified via data from experiments, text mining, or protein networks. A reverse docking approach can find possible protein targets by assuming that ligands with similar structures attach to proteins with comparable affinities, resulting in similar biological effects (Shaker et al. 2021). The molecular docking technique is an advantageous instrument for simulating connections among substances and proteins at the level of an atom. This approach allows for an examination of intracellular dynamics beneath the binding locations of target proteins, thereby providing conclusions about underlying processes in biochemistry (Huang et al. 2003). The multidimensional configuration of a protein's structure is an essential prerequisite in comprehending its workings and connection to evolution. Several investigations have highlighted the promising in silico utilization of homology modeling, to predict 3D structures and discern evolutionary relationships through the analysis of deduced amino acid sequences (Ezat et al. 2014). Moreover, the physicochemical attributes of proteins provide a fundamental comprehension of the stereochemistry inherent in the polypeptide backbone. The Ramachandran map has been firmly recognized as a powerful computational instrument for confirming the stereochemistry of protein structures (Ramachandran et al. 1963). ERRAT software is designed to validate protein structures obtained through crystallography (MacArthur et al. 1994; Kandasamy et al. 2018). Usage of in silico approaches have gained popularity as bioinformatics and proteomics technologies have advanced. Techniques like Computational drug target identification approaches minimize the search scope of experimental targets by transforming information about targets into programming language and executing data processing and analysis. They help research cycle to reduce and lower experiment costs (Liao et al. 2022).

In the current investigation, subtilisin produced previously by the wild *B. subtili*s strain ZK3 was subjected to purification analysis employing ammonium sulphate fractionation followed by two chromatographic techniques namely, anion exchange and gel filtration chromatography to obtain a purified protein. Wild subtilisin was assessed using subtilisin specific assay and was characterized by different biochemical featuressuch aslevel of pH, temperature ranges, surfactants, different metal ions, various inhibitors, substrate specificity and kinetic studies. Further it was validated by applying several bioanalytical techniques like TGA, AFM, CD, ^1^H-NMR to check its thermal stability and other structural characterization. The purified protein was then tested for various environmental and healthcare applications goat skin dehairing, feather degradation and blood clot dissolution, respectively. To elucidate the structural and physiochemical attributes of subtilisin, homology modelling and in silico tools such as ScanProsite, InterProScan, ExpasyProtparam, Fold Unfold were employed. Molecular docking and dynamic simulation wereemployed to study the stability and interactions of subtilisin with 3 specific substrates.

Limited reports exist on the current investigation that details the extensive characterization of wild-type subtilisin by validating it using bioanalytical techniques. Also, computational and *insilico* methodologies utilized to gain insights into the molecular aspects of subtilisin molecule is not much reported. This novelty contributes to the scientific field's knowledge base.

## Materials and methods

### Reagents and media components

Components utilized throughout the current investigation were acquired through Merck and Company., Inc., USA and Sigma Aldrich, Inc. USA Private Limited. HiTrapDEAE FF column for anion exchange chromatography and Sephadex G-100 had been acquired fromCytiva and Sigma Aldrich, Inc. USA Pvt. Ltd. respectively.

Wild subtilisin was produced by *Bacillus subtilis* strain ZK3 using agro-waste substances which was further statistically optimized to obtain highest yield. This subtilisin from the previous study is utilized for its purification and biochemical characterization in the current research (Shettar et al. 2023a).

### Subtilisin (Casein) activity

The enzymatic capability of subtilisin was evaluated by monitoring the discharge of liberated residues of tyrosine throughout casein hydrolysis. An amalgam of 0.5 mL wild subtilisin along with 0.5 mL containing a ten mg/mL casein mixture, created in a 100 mM glycine–NaOH buffered solution at pH 9, underwent incubation for 10 min at 80 ºC. The reaction was interrupted through the inclusion of 2.5 ml of a 10% solution of trichloroacetic acid (TCA) and kept quiescent for thirty minutes at an ambient temperature. The mixture was subsequently spun at 10,000 revolutions per minute over a period of fifteen minutes in order to separate the precipitate. A volume containing 0.5 mL that contained the slightly transparent supernatant had been combined along with sodium carbonate (2.5 mL) and 0.5 mL volume of Folin-Ciocalteu's phenolic product, which is followed by steady growth at ambient temperature over 30 min. The level of optical density was calculated at an absorbance of 660 nm utilizing a UV–Visible spectrophotometer. A single measurement of subtilisin function has been interpreted as a certain quantity of the enzyme capable of releasing one µM of tyrosine every minute under the specified circumstances (Chauhan et al. 2021; Shettar et al. 2023b).

### Subtilisin specific assay

Subtilisin functionality was evaluated employing the synthetically produced peptide (N-Suc-F-A-A-F-*p*NA) bearing inventory code S2628 manufactured by Sigma Aldrich. Briefly, the experimental procedure involved setting up a reaction by combining a blend (200 µL), comprising an enzyme solution (30 µL), synthetic peptide (30 µL), and 20 mM Tris–HCl (pH 7.4) (140 µL), for a duration of 30 min at 37 °C. The total amount of emitted *p*-nitroaniline was estimated using ultraviolet–visible spectrophotometry at 405 nm. Under conventional assay conditions, The smallest amount of the enzyme's action associated with a synthetic peptide is characterized as the volume of enzyme that generates 1 µmol of *p*-NA every minute (Couto et al. 2022; Shettar et al. 2023a).

### Protein content determination

As reported by (Kim et al. 2020), employed a BCA protein measurement package and standardized bovine serum albumin to figure out the whole soluble protein content (mg) of subtilisin. The measurements were conducted on three separate trials, culminating thefindings as average ± standard deviation.

### Topological examination of *B. subtilis* strain ZK3 by atomic force microscopy (AFM)

The microscopic structure of *B. subtilis* strain ZK3 which produced wild subtilisin was detected using AFM instrument by Nanosurf, Switzerland. A glass slide received a deposition of a 10 µL droplet of nanoparticles, and a thin film was subsequently spread across its surface. Afterward, it was left to air dry for a short duration. The sample was positioned utilizing the piezoelectric scanning device, as well as a silicon nitride cantilever with contact phase was applied for examining its outermost layer. The images were analyzed using Easyscan 2 software (Bagewadi et al. 2020; Shettar et al. 2023a).

### Purification of wild subtilisin

The wild subtilisin from previously identified *Bacillus subtilis* strain ZK3 underwent purification through conventionalized purification methods, involving a multi-stage process as described below:

### Partial purification by ammonium sulphate precipitation

The previously produced statistically optimizedwild subtilisin (Shettar et al. 2023a) underwent salt precipitation through slow addition of ammonium sulfate fractionation at80% saturation level, as per standard chart. The precipitated product has been extracted via spinning at 9000 rpm at 4 °C for 20 min (Singh et al. 2018; Chauhan et al. 2021). The pellet underwent dialysis against double-distilled water overnight. The assessment included measuring both activity of enzyme as well as protein determination. The specific activity was quantified as U/mg of protein (Foophow et al. 2022). The portion demonstrating the highest subtilisin activity was then employed for the subsequent purification step, which involved ion exchange chromatography utilizing aHiTrap diethyl-aminoethyl (DEAE) FF column.

### Anion exchange and Gel filtration chromatography using AKTA start

Post ammonium sulfate precipitate, the dialyzed subtilisin was added to a HiTrap diethyl-aminoethyl (DEAE) FF(1 mL) anion exchange chromatographic column. During gradient elution with NaCl in above buffering agent (0–0.5 M), two milliliter fractions were extracted at an average speed of flow of 0.5 mL every minute. The proportion of proteins within the aforementioned sections have been determined through calculating the amount of absorbance at 280 nm, while the activity of enzymes was evaluated employing the specified substrate material.

The protein sample, abundant in subtilisin activity, from DEAE column was consolidated and introduced into the Sephadex G-100 gel filtration column (1.5 × 120 cm). The rate of flow stayed constant at 15 mL per hour, while portions containing a total capacity of 1.25 ml were taken into account every five minutes throughout the experiment. The fraction collection was resumed, while their coefficient of absorption had been determined at 280 nm. The fractions exhibiting highest activity wasregarded as an enzyme that had undergone multi-step purification and was of exceptional quality (Hakim et al. 2018; Lakshmi et al. 2018). The specific enzyme activity (U/mg), yield (%), and purification factor of crude and filtered wild subtilisin have been evaluated utilizing subtilisin-specific assays.

### SDS-PAGE analysis

Wild subtilisin was measured employing the typical SDS-PAGE means established by(LAEMMLI 1970) 0.12% (w/v) resolving gel and 4% (w/v) stacking gel followed by CBB R-250 staining was employed to evaluate the presence of wild subtilisin (Sachin et al. 2021; Chauhan et al. 2021).

### Biochemical assessment of purified wild subtilisin

#### Influence on pH and durability

The impact of pH on subtilisin action was studied, the substrate was formed using multiple buffers with distinct pH levels at 50 °C. The buffer solutions used included 50 mM acetate of sodium solution the pH level 4–5, phosphate buffer with a pH 6–7 (50 mM), plus a buffer of Tris–HCl with pH 7–9 (50 mM) and lastly glycine–NaOH solution spanning pH of 9–13 at 50 mM (Chauhan et al. 2021; Foophow et al. 2022). For exploring pH stability, wild subtilisin underwent preincubation for up to 20 h in the specified buffers of pH 9–11, and its subtilisin activity was subsequently calculated with previously outlined method (Mushtaq et al. 2021a).

#### The influence of temperature and consistency

The influence of temperature on subtilisin function had been investigated throughout a varied spectrum (35–100 °C). Furthermore, the degree of thermal durability was tested via pre-incubating the digestive enzyme itself over 20 h under varied temperatures comprising 50, 60, and 70 °C, and then determining respective % relative actions (Hakim et al. 2018).

#### Effect of different surfactants

The influence of surfactants at levels of 1.0% (w/v) including sodium dodecyl sulfate (SDS), tween-40, the triton X-100 and tween-80, in addition a substance that oxidizes called hydrogen peroxide (H_2_O_2_) at amount equal to 1.0% (v/v) on subtilisin activity was investigated. Each of the reactions performed a 30-min incubation utilizing the specific substrate, as well as their relative activity was subsequently calculated in percentages (Lakshmi et al. 2018; Baykara et al. 2021).

#### Effect of metal ions

To understand the enzyme catalytic effects of diverse ions of metal on the effectiveness of the heat-resistant wild subtilisin, the enzyme had been preincubated in a solution of a concentration of 0.5 M ions made of metal(FeSO_4_, NaCl, CaCl_2_, MgSO_4_, CuSO_4_,KCl and ZnSO_4_). The measurement of enzymatic activity was conducted utilizing N-Suc-F-A-A-F-*p*NA and presented as a percentage of relative activity (Manavalan et al. 2020; Foophow et al. 2022).

#### Effect of additives and organic solvents

The pure subtilisin received treatment for 30 min with several additives at 1 M dosage, including Phenylmethylsulfonyl fluoride (PMSF), Ethylenediaminetetraacetic acid (EDTA), dithiothreitol, and β-mercaptoethanol. Following this, the reaction was commenced, and the subtilisin activity relative to standard conditions was evaluated (Lakshmi et al. 2018; Kim et al. 2020).

The catalytic efficiency was evaluated in a mixture of solvents that are organic at a solution concentration of 10% (v/v)—specifically benzene, methanol, ethyl acetate, ethanol, chloroform and glycerol—was conducted. Following this, the enzyme's relative activity was determined using the optimized assay (Singh et al. 2018).

#### Impact of specificity of substrates and kinetics

The substrate suitability investigation of the pure wild subtilisin comprised examination making use of multiple components, including the casein protein (natural), azo-casein (altered), N-acetyl-L-tyrosine ethyl ester monohydrate (ATEE), N-Benzoyl-L-tyrosine ethyl ester (BTEE) (esters), in addition to N-Suc-F-A-A-F-*p*NA (a synthetic peptide). The responses between wild subtilisin and every substrate followed the previously described standard test protocols. Regarding the azo-casein, a unit of subtilisin action was evaluated as quantity of sample being assessed capable of hydrolyzing azo-casein, resulting in a 0.01 increase in value of absorbance with specified assay circumstances. For the N-Suc-F-A-A-F-*p*NA substrate, a single unit of subtilisin efficiency was determined as the enzyme producing 1 μmol of *p*-nitroanilide (*p*-NA) every minute under prescribed testing circumstances. To identify the BTEE base, a subtilisin performance value (esterase or amidase) had been chosen as the enzyme's quantity, which induced a modification in absorbency at a rate of 0.001 per minute under usual test conditions.

The calculation of kinetic features, encompassing the Michaelis–Menten constant (K_m_), maximum velocity (V_max_), turnover number (k_cat_), and k_cat_/K_m_, was carried out through the utilization of the Lineweaver–Burk plot and equation, following the methodology outlined by (Shettar et al. 2023b). The kinetic constants have been computed by utilizing casein and N-Suc-F-A-A-F-*p*NA as substrates that are available with dosages fluctuating between 0.1 and 10 mM, under classic laboratory conditions (Foophow et al. 2022).

### Bioanalytical assessment of purified wild subtilisin

#### Thermogravimetric analysis (TGA)

Evaluating the thermal stability of the wild purified subtilisin involved conducting TGA analysis using an SDT-Q600 instrument. Heating the 5 mL sample happened in an appropriately controlled nitrogen environment, at an average thermal flow of 10 °C every minute, encompassing an effective temperature that spanned 25–800 °C (Bagewadi et al. 2020).

#### CD

The investigation of the secondary framework of freshly purified wild subtilisin took place under a liquid condition at 25 °C, covering the wavelength range from 190 to 320 nm. The measurement was performed by utilizing a Jasco J-1500 Circular Dichroism spectrum analyzer from Japan which had been purging with nitrogen gas prior to use.The wild original subtilisin was methodically neutralized in a 10 mM buffer solution of sodium phosphate at an equilibrium pH of 7.0 to achieve an end-point dosage of 50 μg/mL.Circular Dichroism spectrum was methodically obtained employing a cuvette made of quartz containing an incident light path size of 1 mm and an imaging rate of 50 nm per minute. The frequencies were taken in CD mdegand translated into molar ellipticity. The in-depth analysis of the protein's 2D structure and helical content was conducted utilizing the advanced online K2D2 software (Golneshin et al. 2020; Shettar et al. 2023b).

#### Nuclear magnetic resonance(^1^H-NMR)

NMR spectroscopy utilizing the Jeol Resonance instrument was deployed for the analysis of wild purified subtilisin through Proton- Nuclear Magnetic Resonance(^1^H-NMR). This assessment occurred at 400 MHz under room temperature conditions, with tetramethylsilane serving as the internal standard. Deuterated chloroform (CDCl_3_) was the solvent utilized for dissolving the processed subtilisin. The ^1^H-NMR spectra's peaks were assigned in compliance with previously published literature, and signals were carefully recorded across 0 and 11 ppm (Shettar et al. 2023b).

### Applications

#### Dehairing activity

The procurement of goat hide took place at a nearby market, and it underwent a thorough cleansing process using distilled water. Small segments of the hide, measuring 4 × 4 cm^2^, were immersed in the purified wild subtilisin and 1% sodium sulphide for incubation at 40 °C in shaker incubatorat 150 rpm for a duration ranging from 4 to 24 h.After the incubation interval, the segments were purified with distilled water, and the elimination of detached hairs ensued. Following this intervention, the effectiveness of the hair removal process was appraised by examining attributes such as color, the general look of leather, and duration necessary for effortless hair removal off hide post-treatment was calculated (Hakim et al. 2018; Manavalan et al. 2020).

#### Blood clot dissolution

To fill centrifuge tubes, 0.5 mL of sheep blood was procured from a neighboring butcher shop. Two distinct tubes comprising blood were subjected to treatment with distilled water as a means of control and wild subtilisin as a test agent, furthermore, the dissolution of the clot was found. During the methodology, the efficacy of subtilisin was evaluated using an uninterrupted incubation duration of 4 h. Following a 4 h incubation at 37 °C, an analysis was conducted on the samples to determine the percentage of total weight dissociation. After centrifuging the tubes at 20,000 rpm for twenty minutes, the weights of both the centrifuge tubes and pellet were measured (Sharma et al. 2019; Li et al. 2021). The clot dissolution rate was evaluated as follows:

$$ {\text{Clot dissolution rate}}\, = \,\frac{{{\text{Clot weight prior dissolution}}{-}{\text{clot weight post dissolution}}}}{{\text{clot weight prior dissolution}}}\, \times \,100\% $$

#### Feather degradation

Acquired from a poultry farm, whole chicken feathers received an extensive wash utilizing faucet water in order to eliminate any remnants of blood, succeeded by a meticulous rinse using water which is distilled and autoclaving. Subsequently, those feathers had been permitted to dry out in the air overnight. The process of disintegration for the full chicken feather was then performed by soaking it beneath the refined enzyme at 60 °C for approximately 4 h (Mushtaq et al. 2021a).

### *In-silico* analysis

#### Sequence analysis and GenBank submission

The 16S rRNA of the potential isolate ZK3 underwent sequencing, following procedures outlined in a prior research by (Shettar et al. 2023a). Similarity analyses was subsequently performed utilizing the BLAST tools available through the National Center for Biotechnology Information (NCBI) website (http://www.ncbi.nlm.nih.gov/blast), operated by the United States. The phylogenetic tree recognized it as *Bacillus subtilis* strain ZK3, and the resultant genetic sequence was officially filed with GenBank having accession numbers OQ300503.1. This wild strain possesses the capacity to generate subtilisin molecule.

#### Anticipation of functional sites and identification of protein family

As previously indicated in (Shettar et al. 2023b), the gene sequence generated from *B. subtilis* strain ZK3 was converted into a protein sequence applying the Expasy translate program (https://web.expasy.org/translate/). The gene and protein sequences underwent alignment and similarity analysis through the utilization of the NCBI BLAST tool and the ClustalW multiple aligner. The tree of phylogenetic relationships was constructed employing MEGA 11 software, thereby applying the neighbor-joining strategy to determine those with the most closely associated sequences. The protein databases offered by the Expasy-PROSITE, accessible at https://prosite.expasy.org/, are fundamental in differentiating protein categories, families, operational sites, as well as correlated patterns and characteristics. The subtilisin's enzymatic motif and active regions have been predicted with ScanProsite (https://prosite.expasy.org/scanprosite/). InterProScan (https://www.ebi.ac.uk/interpro/) as part of EMBL-EBI (https://www.ebi.ac.uk/) conducts analysis of proteins by categorizing them into families and forecasting domains and crucial sites (Mechri et al. 2021; Mahmoud et al. 2021).

#### Physiochemical characterization of wild subtilisin

The ExPASy service's ProtParam assessment tool (https://web.expasy.org/protparam/) was utilized for estimating chemical and physical attributes involving molecular mass, theoretical value of isoelectric point (pI), amino acid organization, instability index, aliphatic index, and grand average of hydropathy (GRAVY) (Mahmoud et al. 2021; Almahasheer et al. 2022).

#### Homology modelling

The translated protein sequence given below was utilizedto build model by employing automated model prediction tool SWISS-MODEL (https://swissmodel.expasy.org/).

MRSKKLWISLLFALTLIFTMAFSNMSAQAAGKSSTEKKYIVGFKQTMSAMSSAKKKDVISEKGGKVQKQFKYVNAAAATLDAKAVKELKQDPSVAYVEEDHIAHQYAQSVPYGISQIKAPALHSQGYTGSNVKVAVIDSGIDSSHPDLNVRGGASFVPSETNPYQDGSSHGTHVAGTVAALNNSIGVLGVAPNASLYAVKVLDSTGNGQYSWIINGIEWAISNKMDVINMSLGGPSGSTALKSVVDRAVASGIVVVAAAGNEGTSGSSSTIGYPAKYPSTIAVGAVNSSNQRGSFSSVGPELDVMAPGVSIQSTLPGGTYGAYNGTSMATPHVAGAAALILSKHPTWTNAQVRDRLESTTTYLGNSFYYGKGLINVQAAAQ*

The model representing GMQE score > 0.8, highest sequence identity and coverage was selected for further analysis. The resulting template 3WHI crystal structure of *Bacillus subtilis* subsp. *subtilis* str. 168 was saved in PDB format. Thevisualization and analysis of three-dimensional model were conducted using the PyMOLv2.3, available at http://www.pymol.org (Mechri et al. 2021).MolProbity tool (http://molprobity.biochem.duke.edu/) was utilized to study the statistical distribution of the Ramachandran plot (Almahasheer et al. 2022). The template of choice was subsequently utilized for additional study. The level of precision of developed protein framework model was evaluated via assessment of the stereochemical features deploying the SAVES server v6.0 (https://saves.mbi.ucla.edu/) including ERRAT, VERIFY 3D (Bowie et al. 1991; Lüthy et al. 1992) and PROCHECK (Laskowski et al. 1993). The highly flexible regions of the subtilisin were identified using the FoldUnfold (https://bio.tools/foldunfold) tool.

#### Molecular docking of subtilisin with different substrates

The set of ligands namely N-Suc-F-A-A-F-*p*NA, N-acetyl-L-tyrosine ethyl ester monohydrate (ATEE) and N-Benzoyl-L-tyrosine ethyl ester (BTEE) (esters), acquired through substrate specificity from biochemical characterization of subtilisin, the structures of molecules were retrieved in.sdf format from PubChem. Following that, the.sdf files of ligands were transformed into pdb format usingPyMOL (http://www.pymol.org) and saved. The docking of protein and ligands was performed using AutoDock Tools v1.5.7 (Rekik et al. 2019). Dragging and dropping pdb file of protein 3WHI into Discovery Studio software (https://www.3ds.com/products/biovia/discovery-studio), facilitated acquisition of sphere object attributes in which the water molecules were deleted and all ligands were unchecked. Once the sphere appears on the protein, its sphere object attributes of x = −45.913, y = 51.057, z = 1.431 was noted down. Next, in the autodock tools, the protein.pdb file was loaded and serially options were followed to prepare the protein. Within the editing option, water molecules were eliminated, polar hydrogen atoms were incorporated, and Kollman charges were applied. A config.txt file was created by adding values of the x, y, z attributes, size of x, y, z = 20, energy range = 4 and exhaustiveness = 8 and saved the file. Finally, using Autodock vina, output. pdbqt and log, txt files were generated which depicts the results of the protein with each ligand. AutoDock was performed for each ligand with the same protein (Yaraguppi et al. 2021). Proceeding with further molecular dynamic simulation analysis, only the pose that demonstrated accurate interaction to the target site and had the lowest binding energy scorewas utilized.The analysis of binding propertiesamong the target protein and selected ligand that has formed the complex obtained from the docking study was evaluatedemploying the Discovery studio software (Bagewadi et al. 2023).

#### Molecular dynamic simulation

The Molecular Dynamics study selected the subtilisin and N-Suc-F-A-A-F-*p*NAcomplex with the highest docking score. To assess the stability of subtilisin in relation to the synthetic substrateN-Suc-F-A-A-F-*p*NA, The GROMACS software(v 2019.4) was utilized to perform Molecular Dynamics Simulations. The study utilized the Gomocs54a7 force field (Revankar et al. 2023). The acquisition of Ligand topology was facilitated using a prodrug server. The Molecular Dynamics (MD) simulations had been designed to last over 200 ns whilst adhering to a constant temperature of 300 Kelvin integrating the Berendsen thermostat. Moreover, the computer programs were run at an uninterrupted pressure level of 1.01325 bars, mimicking the naturally occurring temperature as well as pressure parameters that lie within the human body (Yaraguppi et al. 2022; Revankar et al. 2023). Subsequently, the establishment of the complex system comprised an inclusion of water in addition to ions to harmonize the environment, that encompassed sodium (Na^+^) opposing ions. The system underwent energy minimization using the steepest descent method.The Isothermal-Isobaric (NPT) ensemble was utilized, incorporating a simple point charge water model within a 10 Å orthorhombic box (Yaraguppi et al. 2021). The Gromacs Package is employed for the analysis of data derived from the dynamics of the protein-substrate complex. Calculations are executed upon the atoms that are within the protein framework, alongside the ligand coupled together with the protein, in order to evaluate the Root Mean Square Deviation (RMSD).Throughout the Molecular Dynamics simulation, various additional factors, such as Radius of Gyration (Rg), Solvent Accessible Surface Area (SASA), and hydrogen bond analysis, were also considered (Rekik et al. 2019).To compute and examine these parameters, the following utilities are utilized: gmx-rms (for RMSD), gmx-rmsf (for RMSF), gmx-gyrate (for Rg), gmxhbond (for Hydrogen bond), and gmx-sasa (for SASA). The xmgrace program was utilized to produce graphical representations based on the simulation findings (Revankar et al. 2023).

#### Molecular mechanics poison surface area analysis (MMPBSA)

The MMPBSA approach has been utilized to assess the substance's binding independent energy (ΔG binding). To determine interaction potential of drug molecules within the stable structure of the simulation, the open-source tool g_mmpbsa is utilized. Calculations are carried out using the GROMACS utility g_mmpbsa tool, available at https://rashmikumari.github.io/g_mmpbsa/. Leveraging the tool mentioned earlier, the MMPBSA technique is utilized for the assessment of diverse binding energy components. This requires assessing the amount of entropy involvement and energy participation of particular amino acids. The estimate of ΔG takes into account the conclusive 50 ns that comprise the trajectories coming from simulations. This specific duration is chosen as it represents a stable phase in the simulation. The MMPBSA calculation carried out on this region supplies further evidence to corroborate the findings of the complex simulation.The disparity among the protein's conformations in the existence or absence of the ligand serves to figure out the inhibitor's unattached relationships potential around the protein. The following formula is utilized.

ΔG binding = G-complex—(G-protein + G-Ligand) (Yaraguppi et al. 2022; Revankar et al. 2023).

### Statistical analysis

For every assessment, not less than three distinct, repetitions were performed. The average (mean ± SD) and mean of the recurred findings conveyed outcomes of experiment.

## Results and discussion

### Atomic force microscopy (AFM)

**Fig. 1:**
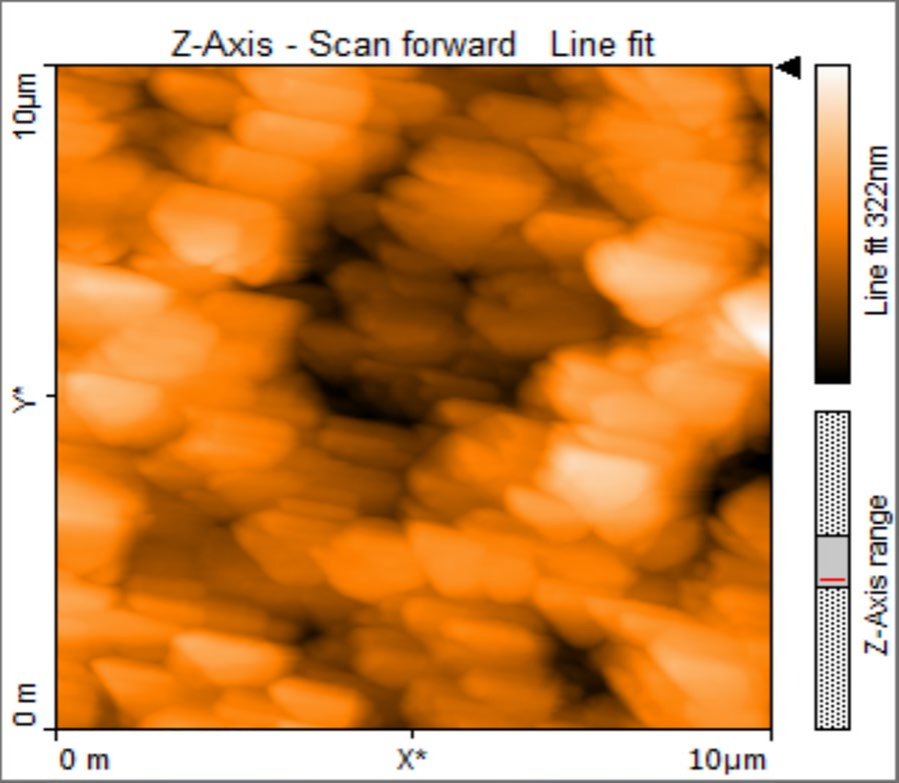
Atomic Force Microscopic image of *B. subtilis* strain ZK3

AFM enables researchers to capture pictures with excellent resolution and elevation profiles by meticulously sweeping a sharp point all over the outermost portion of the sample. This methodology allows for the visualization of the topological surface of the studied strain and its clustering patterns, contributing to a comprehensive characterization of its physical properties. Connected to a flexible cantilever, when the AFM tip comes into connection with the sample surface, it causes the cantilever to bend due to attraction and repelling forces exerted by atoms and molecules that are on the tip's outer surface (Shettar et al. 2023a).The cantilever's deflection is then monitored, and a mechanism called feedback operates to guarantee that the tension across the point of the cantilever and the object being studied remains consistent. Here in this investigation, the morphology of the identified *B. subtilis* strain ZK3 which produced wild subtilisin is being studied. The micrograph displays rod-shaped bacteria known as *Bacilli*, exhibits a surface characterized by its smooth texture. The formation of ridges is likely attributed to the folding of the coat caused by variations in spore size upon the process of dehydration (Fig. 1).

Wild subtilisin is being reported to being used in various industrial as well as biotechnological applications making it a potent molecule. As noted in earlier AFM investigations, the notable surface attributes of air-dried *B. subtilis* wild-type spores encompass surface ridges that extend parallel to the spore's long axis (Boudreau et al. 2023). The spores exhibited an overall ellipsoidal shape. Among the specimens, *B. cereus* displayed the most substantial spore population affixed to the soft graphite exterior, followed by *B. anthracis, B. globigii*, and *B. thuringiensis* (Tachibana et al. 2021)*.* In a recent research work to optimize and enhance the functional properties of rapeseed meal using a mutant *B. subtilis* strain displayed that post 92 h of fermentation, *B. subtilis* morphology and surface topography changed significantly, according to SEM and AFM outcomes (Betchem et al. 2023).

### Purification of wild subtilisin

#### Ammonium sulphate precipitation of wild subtilisin

Serine proteases constitute an enzyme class with pivotal involvement in diverse biological processes, specifically in regulating cellular functions and facilitating digestion. Subtilisin is a serine protease performing the similar functions and in the present study, the wild subtilisin which was previously produced by *Bacillus subtilis* strain ZK3, using raw agro-waste components was statistically optimized to produce high yield (Shettar et al. 2023a). The crude protein is now being used for further purification and biochemical characterization. The liquid portion obtained post centrifugation, known as supernatant, is crude. It was subsequently subjected to precipitation using ammonium sulfate at 80%. Notably, the most effective percentage for precipitating the crude wild subtilisin was found to be 80% saturation. The enzyme precipitate obtained underwent further purification through dialysis. The initial specific activity of the crude wild subtilisin, at 285.185 U/mg, experienced an augmentation to 340 U/mg through ammonium sulfate precipitation, and further increased to 391.304 U/mg after dialysis, yielding a purification fold of 1.372 with an approximately 34.074% yield as shown in Table 1.

**Table 1:** Overview of the purifying phases of wild subtilisin

Purification step	Total activity (units)^ab^ X 10^3^	Total protein (mg)^ac^	Specific activity (U/mg of protein)	Activity yield (%)	Purification fold
Crude	77 ± 19	270 ± 15	285.185	100	1
Ammonium sulphate saturation (80%)	51 ± 15	150 ± 13	340.000	55.556	1.192
Dialysis	36 ± 10	92 ± 8	391.304	34.074	1.372
DEAE-anion exchange	16.5 ± 12	31.5 ± 7.2	523.810	11.667	1.837
Gel filtration	11 ± 9	9.5 ± 6	1157.895	6.333	3.406

^a^Values reported are the average ± standard deviation of three studies

^b^Under conventional experimental settings, a single measure of subtilisin activity represents the enzyme's ability to hydrolyze casein substrate and create 1 µM of equivalent tyrosine every minute

^c^Protein concentration was measured utilizing the Bradford technique

The investigation into ammonium sulfate precipitation for alkaline protease produced by *Bacillus alcalophilus* LW8 indicated high yield of 78.53%, however the specific activity (225 U/mg) is notably lower compared to the wild subtilisin, which exhibited 340 U/mg and purification fold (1.21) is similar to the present study showing 1.192. Whereas the purification fold of dialysate of wild subtilisin (1.372) is higher than that of alkaline protease (1.24) (Rathod and Pathak 2014). The OM-5 alkaline protease generated by *Nocardiopsis alba* strain OM-5was similarly precipitated using ammonium sulfate between 30 and 70% saturation, resulting in a total protein content of 22.66 mg while the wild subtilisin showed higher at 150 mg. The yield obtained for the current study (55.55%) shows slight difference with the yield of OM-5 alkaline protease of 58.10% (Chauhan et al. 2021). A recent study revealed that *Streptomyces violaceoruber* produced alkaline protease, which had 5.2 U/mL activity at 60% ammonium sulphate. The precipitated alkaline protease fraction was refined using ion-exchange and gel filtration column chromatography after being dialyzed overnight at 4 °C (Al-Dhabaan 2024). A thermostable protease was largely purified using ammonium sulphate precipitation and dialysis at 70–80% with 1.56-fold improvement in purity. While dialyzed protease efficiency was enhanced to 1.35 times (Majeed et al. 2024).

#### Multi-step purification of wild subtilisin

**Fig. 2:**
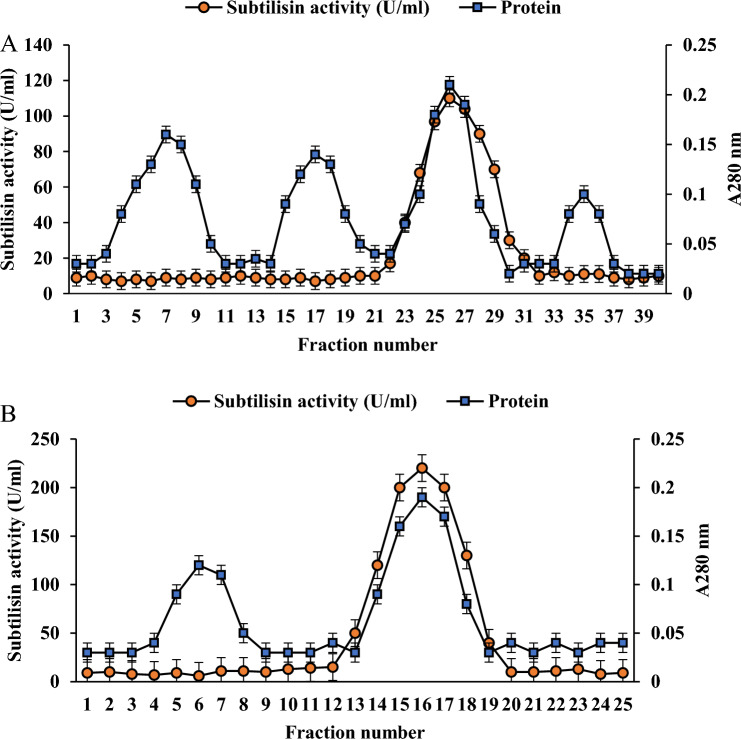
Protein profile by AKTA start- (**A**) DEAE-anion exchange chromatography (**B**) Gel filtration chromatography

The dialyzed sample acquired in the preceding step was introduced onto an anion exchange chromatography with HiTrap diethyl-aminoethyl (DEAE) FF column, and the elution process was subsequently conducted. This gave specific activity of 523.810 U/mg, 11.667% yield and an increased purification fold of 1.837 (Table 1). This portion underwent elution with active fractions eluted using20 mM NaCl (Fig. 2A). The wild subtilisin preparation obtained through anion exchange chromatography was subsequently put through gel filtration chromatography utilizing a Sephadex G-100 column. This enhanced the purification fold to 3.406 as shown in Table 1. The fractionation pattern of Sephadex G-100 chromatography is depicted in Fig. 2B. The purification table demonstrated the extent of purification at each phase (Table 1).This purified wild subtilisin was further used for molecular weight determination.

The refinement of wild subtilisin is crucial to advance our comprehension of the enzyme's operation. Several different chromatographic techniques like ion exchange, gel filtration, affinity are used to purify the serine proteases (Lakshmi et al. 2018). The current study of wild subtilisin has shown higher purification fold of 1.837 at the DEAE-anion exchange chromatographic step and lower yield of 11.667% comparatively. The yield of alkaline protease from two different strains namely, *B. subtilis* AKAL7 and *Exiguobacterium indicum* AKAL11 post DEAE-cellulose chromatography was examined to be 9.75% and 9.41% which lesser than the current study (11.667%) (Hakim et al. 2018). (Manavalan et al. 2020) documented the application of DEAE-cellulose and sephadex G-100 column chromatography to purify an alkaline protease, resulting ultimately in a 2.45-fold enhancement in the protein's purity and specific activity of 897 U/mg which is relatively lower than the current study of wild subtilisin. Recently, in an investigation by (Sachin et al. 2021), specific activity of a serine protease (CmSP) purified by seeds of *C. maderaspatensis* post gel filtration chromatography, depicted activity of 884.6 U/mg while wild subtilisin displayed 1157.895 U/mg. The Äkta Purifier FPLC instrument was used to purify serine protease. In the initial phase, the HiTrap DEAE FF was connected to the FPLC which yielded a purification fold of 1.8. The second purification stage involved using a Superdex 75 10/300 G L molecular exclusion matrix. The FPLC purification techniques yielded high purity levels (Gomes et al. 2020). The alkaline protease enzyme originated from *B. pumilus* Y7 culture medium was purified by single-step hydrophobic interaction chromatography. The technique resulted in a 5.4-fold purification and 52.8% recovery yield compared to the initial crude extract (Duman and Tekin 2020). Crude protease activity was measured at 0.63 mg/mL of protein and 111.56 U/mg specific activity. Protein concentrations of 0.57 mg/mL and 0.44 mg/mL were found using 70% ammonium sulphate saturation and gel chromatography, respectively, exhibiting specific activity of 143.65 U/mg (Shad et al. 2024). Ethanol precipitation for a serine peptidase from *A. terreus*was studied, achieving a 2.5-fold purification factor with 70% ethanol. This method outperformed the chromatography techniques used in subsequent steps (Biaggio et al. 2016). OM-5 is a thermostable alkaline serine protease which was purified using hydrophobic interaction chromatography (HIC) method with 5.01 purification fold (Chauhan et al. 2021).

#### Molecular weight determination

**Fig. 3:**
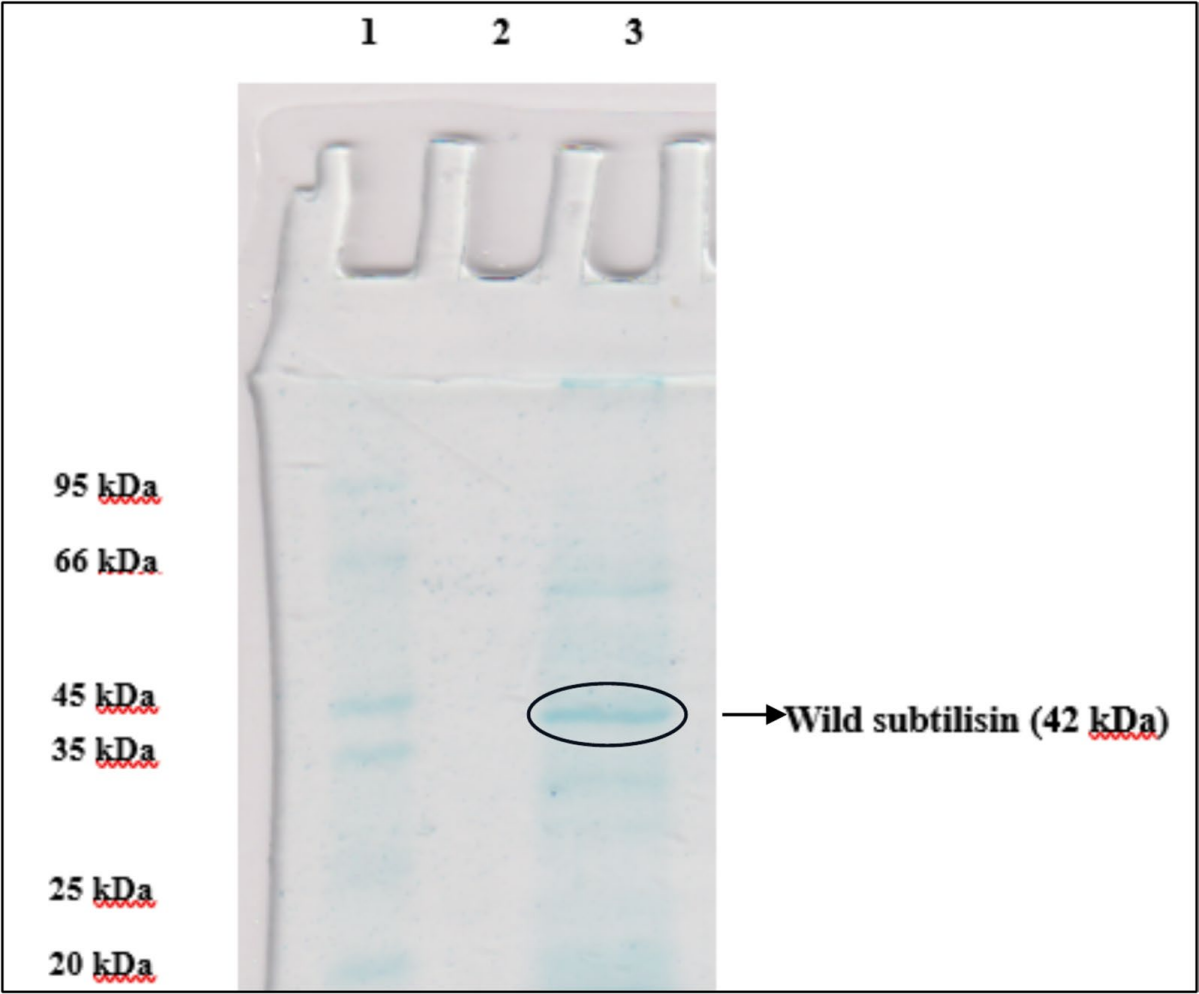
SDS-PAGE of wild subtilisin

The pure wild subtilisin's molecular weight was determined using the denaturing and reducing SDS-PAGE procedure, which revealed it to be around 42 kDa. A mid-range marker covering proteins between 14 and 95 kDa was employed to evaluate the molecular mass of wild subtilisin, as illustrated in the Fig. 3.

The anticipated weight of partially pure protease from *B. subtilis* was 40 kDa, while that from *E. indicum* was predicted to be 70 kDa (Hakim et al. 2018). A research work by (Kim et al. 2020), SDS–PAGE suggested that the molecular masses of two fibrinolytic enzymes were closest 36 kDa (Neog et al. 2024). Proteins having molecular masses from 6.5 to 62 kDa were found in the chihuil enzyme extracts. Protein bands disappeared during semi-purification due to fractions of protein removal, however a 29 kDa protein band remained across the process (Rios-Herrera et al. 2020). Another study reported ~ 33 kDa purified protease isolated from *B. megaterium*-TK1, further confirmed by zymographic studies (Manavalan et al. 2020). In recent work, alkaline protease with a band of 45 kDa was obtained, produced from *Streptomyces violaceoruber* was electrophoresed (Al-Dhabaan 2024). A researcher studied the molecular weight of alkaline serine protease from *B. cereus strain* S8 found to be 22 kDa which was confirmed by gel filtration chromatography (Lakshmi et al. 2018). The molecular weight of a thermostable protease was estimated to be 65 kDa by SDS-PAGE, Native-PAGE, casein-zymography, and size exclusion by HPLC (Omrane Benmrad et al. 2019).

### Biochemical characterization of wild subtilisin.

#### pH effect and its stability.

**Fig. 4:**
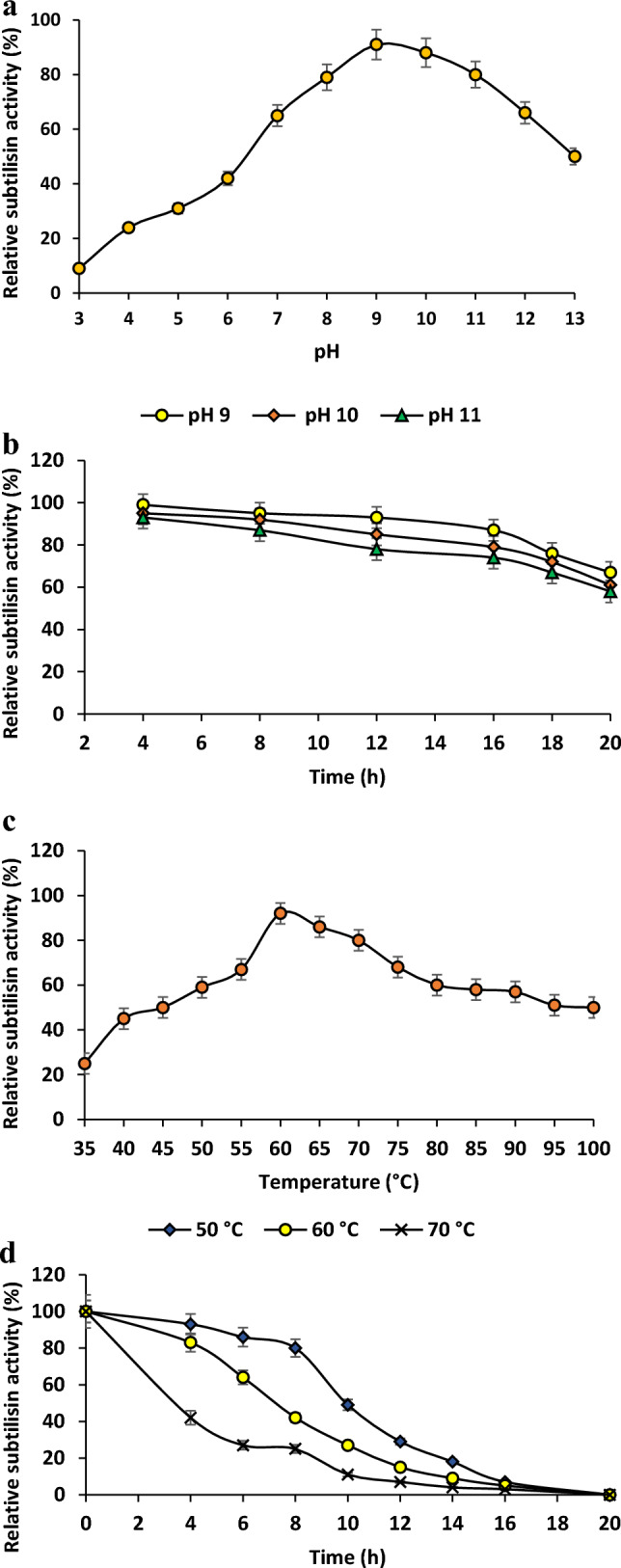
Biochemical characterization of wild subtilisin (**a**) pH effect, (**b**) pH stability, (**c**) temperature effect (**d**) temperature stability

Wild subtilisin's competency was examined at 50 ℃ in a pH interval from 3 to 13 making use of casein as the base material. The wild subtilisin demonstrated substantial activity in the alkaline pH spectrum, notably between 9 and 11, sustaining over 80% in terms of relative efficiency. The optimal pH was observed at 9, as illustrated in Fig. 4A. Commencing in an acidic pH range of 3–6, a value less than half of the relevant performance was recorded. The drop in function under an acidic circumstance could possibly be related to shifts in ionic states of enzyme's locations of activity, thereby impacting the structural and folding attributes of proteins.Nonetheless, among the elevated pH (alkaline) levels of between 12 and 13, the corresponding performances were found to fall in the spectrum between 50 and 66%.The endurance of wild subtilisin against varying pH conditions was explored through pre-incubation at pH level 9, 10, and 11, maintaining at 50 ℃ for a time span of 20 h. Wild subtilisin demonstrated exceptional durability at pH level of 9until14-hour period, retaining over ~ 90% of its relative activity, as illustrated in Fig. 4B predicting its alkaline properties. While the stability remained upto12 h and 8 h for pH 10 and pH 11 respectively with > 92% activities.

The protease generated by *B. subtilis* K-1 (BSK-1) demonstrated significant enzymatic activity under alkaline conditions, showing robust performance at pH 8.0 and 9.0 while it maintained its remarkable stability in the pH span of 7–10 (Singh et al. 2018). The results from current work obtained were familiar with the ideal pH for fibrinolytic activity of JP-I is 8.1, while for JP-II, it is 9.9 from (Kim et al. 2020). The performance of JP-I and JP-II maintained steady from 5 to 10.5 pH level, suggesting the enzymes maintain durability over a wide pH spectrum. Another study goes in line with the wild subtilisin’s pH properties which said that protease from *B. subtilis* and *E. indicum* displayed significant activity across a wide pH spectrum, 7.0–12.0, with peak activity at pH 9.0 and also displayed stability between pH 7–11 (Hakim et al. 2018). The serine protease performed best at pH level 8 as well as 60 °C. It demonstrated significant inhibition by PMSF, confirming that it belonged to serine protease category (Neog et al. 2024). The effectiveness of crude protease was investigated at 6–11 pH, and discovered that protease continued to be functional in the pH levels 9–10, with a maximal efficiency of 72.17 U/mL at the pH level 10.0 (Shad et al. 2024).

#### Temperature effect and its stability

The purified wild subtilisin preserved its functionality at temperatures ranging from 45 to 100 °C with > 50% relative activities, as illustrated in Fig. 4C. It was established that the ideal temperature for its effect to occur occurred 60°Cat pH 9, exhibiting 92% relative activity, signifying its nature as an alkali-thermostable enzyme.Exploration of thermostability occurred over for 20 hfor a temperature span of 50–70 °C. The half-life periods for wild subtilisin at 50, 60, and 70 °C were established as 8, 6, and 2 h, respectively, as depicted in Fig. 4D.

Consistent with our current investigation, (Baykara et al. 2021) similarly documented the thermostable characteristics of serine protease from *Geobacillus* sp. GS53 demonstrating robustness at increased temperatures spanning from 25 to 75 °C, with peak activity at 55 °C. In line with our study, the purified halo protease Vpr from *B. licheniformis* KB111 (KB-SP) protease displayed optimum temperature at 50 °C and approximately 60% and 70% of its peak activity at 30 and 60 °C, correspondingly (Foophow et al. 2022). However, examinations into thermal stability of 50–70 °C revealed enzyme's resilience, even at lower temperatures, especially in the context of a high-potential protease produced by *B. amyloliquefaciens*strain HM48 (Mushtaq et al. 2021a). The extracellular serine protease displayed great stability throughout a 5–10 pH range in addition to temperatures reaching 40 °C for 24 h, allowing it to be used in procedures that do not need energy-intensive heating (Gomes et al. 2020).Baweja et al.(Baweja et al. 2016) found that APase performed well at both 50 °C and 4 °C. The capacity of crude protease by *Bacillus subtilis* K-5 was examined from 25 to 75 °C, having 96.32% at 60 °C (Shad et al. 2024).

#### Impact of various surfactants

Furthermore, the resiliency of wild subtilisin was consistently maintained in the presence of an array of anion-based and non-ionic surfactants that were tested, comprising Tween 40, Tween 80, and Triton X-100, all at 1% dosages. Wild subtilisin maintained its effect at approximately 77%, 87%, and 89%, respectively (Table 2). Despite being an anionic surfactant, SDS has been documented to negatively impact protease enzyme activity in some studies (Mushtaq et al. 2021a). However, in the enzymes under the current investigation, 92% of activity was retained even with SDS (1%).The stability was further assessed through incubation with H_2_O_2_, and it retained 80.5% of its activity (Table 2). Generally, proteases demonstrate robustness in various detergent components, though their stability may be compromised in the presence of bleaching agents. However, the purified wild subtilisin showcased remarkable resilience and activity when exposed to surfactants, bleaching agents, and assorted chemical compounds. These attributes are pivotal for its potential application in detergent formulations and various industrial uses.

**Table 2:** Effect of surfactants, metal ions, additives, organic solvents and substrate specificity on purified wild subtilisin

	Factors	Relative subtilisin activity (%**)**
		Wild subtilisin^a^
Control		
	Without treatment	100
Surfactants		
	Triton X-100	89.61 ± 0.4
	SDS	92.20 ± 0.8
	Tween 40	77.92 ± 1.0
	Tween 80	87.01 ± 0.1
	Hydrogen peroxide	80.51 ± 0.3
Metal ions		
	ZnSO_4_	96.10 ± 1.2
	CuSO_4_	99.35 ± 0.7
	FeSO_4_	59.74 ± 0.2
	MgSO_4_	90.90 ± 1.1
	KCl	81.81 ± 0.9
	CaCl_2_	85.71 ± 0.2
	NaCl	79.22 ± 0.5
Additives		
	PMSF	45.45 ± 0.8
	dithiothreitol	84.41 ± 0.6
	β-mercaptoethanol	86.36 ± 0.4
Organic solvents		
	EDTA	90.90 ± 0.1
	ethyl acetate	87.01 ± 1.5
	ethanol	90.90 ± 1.2
	benzene	88.31 ± 0.8
	glycerol	77.92 ± 0.3
	chloroform	80.51 ± 0.7
Substrate specificity		
	methanol	93.50 ± 0.9
	Casein	97.40 ± 0.6
	Azo-casein	96.75 ± 0.3
	ATEE	92.20 ± 0.4
	BTEE	93.50 ± 0.9
	N-Suc-F-A-A-F-*p*NA	98.70 ± 1.1

The relative activity lacking any surfactants, metal ions, additives, or organic solvents was adopted as the control

Every data value denotes mean ± standard deviation

Influences were analyzed for 2 mg/mL enzymes, 1% surfactants, 0.5 M metal ions, 1 M additivesand 10% organic solvents for 30 min

In existing literature, there have been recent accounts discussing the influence of surfactants on protease functionality, affirming their compatibility with detergent applications. As per a particular study, H_2_O_2_ slightly diminished the action of the thermostable protease from *Geobacillus* sp. GS53, known for its effectiveness as a detergent additive, to 81%. Conversely, Triton X-100, Tween 20, Tween 80 and SDS elevated its activity to 98%, 95%, 91% and 83% respectively (Baykara et al. 2021).Therefore, a notable feature of this wild subtilisin lies in its compatibility with tested surfactants and oxidizing agents. It is noteworthy that only a minimum scale of wild microorganisms are known to exhibit compatibility, especially with SDS and oxidizing agents like H_2_O_2_ (Kamran et al. 2015; Mushtaq et al. 2021a). A related study by (Kamran et al. 2015) explained the impact of various surfactants on serine alkaline protease from alkaliphilic *Bacillus* sp. Tween-80 has displayed the relative activity with 80% while wild subtilisin has shown 87%. Another research work on protease named SH21 extracted from *Bacillus siamensis*CSB55 was tested for influence of surfactants on its activity stating that SDS, Tween-80 and Triton X-100 exhibited relative activities of 85%, 88% and 92% respectively resembles with the current study of wild protein (Tarek et al. 2023). The surfactants SDS and CTAB suppressed thermostable alkaline protease’s activity, while Tween-20 and Triton-X 100 marginally increased it (Manavalan et al. 2020).

#### Effect on metal ions

The subtilisin effectiveness of the wild subtilisin was enhanced by all tested metallic ions, with maximum stimulation observed with 0.5 MCuSO_4_ ions at 99% followed by ZnSO_4_ and MgSO_4_ at 96% and 91% respectively (Table 2). This outcome indicates that the enzyme functions optimally when magnesium, copper and zinc are present.The enzyme activity remained unaltered in the abundance of CaCl_2_ (85%) and KCl (81%), while it was inhibited by NaCl and FeSO_4_with 79% and 59% respectively, as displayed in Table 2, at an equivalent concentration to the other metallic ions.The elevation in subtilisin activity with MgSO_4_implied that the metallic ion provided a shielding impact on preventing denaturation. This phenomenon enables the enzyme to maintain its activity even at elevated temperatures (Donaghy and McKay 1993; Kumar and Takagi 1999).

A review of the literature elucidated the influence of cations and demonstrated the highest activity with Mg^2+^ while others remained less. It demonstrates that wild subtilisin, which has also demonstrated comparable action against MgSO_4_ and CaCl_2_, is well-suited for cleansing in water that has hard conditions, when multiple cation molecules like magnesium as well as calcium are available (Kotb et al. 2023). The literature extensively records the inhibitory impact of heavy metallic ions. Similar to the current investigation, the enzyme action of purified protease was positively affected by Cu^2+^, Mg^2+^, succeeded by Mn^2+^ and Ba^2+^, that showed a slightly activity reduction. However, in contrast to ours, Zn^2+^ions slightly reduced the enzyme activity (Lakshmi et al. 2018). This is also similar to our research where wild subtilisin was produced using agro-residues by statistical optimization as cited previously (Shettar et al. 2023a). The serine protease was more activated at 5 mM concentrations of Mn^2+^, Mg^2+^, and K^+^ ions, but it was significantly inhibited by Hg^2+^, Ag^+^, and Co^2+^ ions. It explains that cations have beneficial influence upon proteolytic function help maintaining the enzyme's active region and increase thermal stability (Gomes et al. 2020). Adding K^+^, Ca^+2^, and Mg^+2^ greatly improved activity in Chihuil semi-purified proteases extract (SPE). In contrast, Cu^+2^ and Fe^+2^were found to reduce enzyme activity from its starting levels. Cu^+2^ ions may induce a reduction in proteolytic activity due to their affinity for sulfhydryl groups, potentially inactivating some functioning proteins (Rios-Herrera et al. 2020).

#### Influence of assorted additives and organic solvents

Compounds known as enzyme inhibitors engage with enzymes, resulting in a temporary or enduring impediment to the pace of enzyme-catalyzed reactions or disturbing the normal functionality of enzymes.The impacts of various additives and protease inhibitor are consolidated in Table 2. The fact that PMSF inhibited subtilisin activity by 45% led to the conclusion that the pure wild subtilisin pertains to serine-containing protease group. PMSF operates as a covalent modifier at the active site of enzyme, thereby suppressing every serine protease. As per the existing literature, proteases having serine residue make up almost 1/3rd of all proteases, and the protein under investigation in this study belongs to this particular family (Park et al. 2013). Additional inhibitors, including DTT, did not demonstrate muchinhibition on subtilisin activity (84%).Moreover, β-mercaptoethanol exhibited almost no significant impact on the protein’s activity displaying 86.3%.EDTA, inhibitors of metalloproteases through chelation, decreased subtilisin activity to 90%, upon addition to the purified wild subtilisin (Table 2). It is suggested that they might act as cofactors in this context.The enzyme's resistance to chelators would be a favorable characteristic, especially considering their utilization in detergent formulations as softening of water and removers of stain (R. et al. 2002; Tarek et al. 2023).

A comparable observation regarding the inhibition of alkaline serine protease by PMSF has been previously demonstrated by (Kamran et al. 2015). Similarly, EDTA could not inhibit the protease activity when compared to ours. Another study depicted the inhibition of PMSF to be ~ 42% when treated with alkaline protease from *Bacillus* sp. Mar64. In contrast, EDTA showed suppressed activity (Kotb et al. 2023).The inhibitory impact of EDTA was observed in which the enzyme activity was 91.21% at a 1.0 mM EDTA concentration, but it decreased at higher concentrations as reported by Mushtaq et al. (2021a). An investigation of protease inhibitors, serine protease was totally hindered by PMSF, indicating presence of a serine in active location and implying the aforementioned protease is related to the serine protease family. β-mercaptoethanol did not promote it, but rather inhibited it by 32% (Gomes et al. 2020).

The impact of organic solvents showed a marginal effect on the activity, with methanol exhibiting 93.5% activity retention followed by ethanol (90.9%). In contrast, benzene (88%) and ethyl acetate (87%) had a moderate effect on the wild subtilisin as shown in Table 2. The wild subtilisin maintained its relative activity at 80.5% when exposed to chloroform. Wild subtilisin showcased superior stability in organic solvents, surpassing other proteases from microbes originated by *Bacillus* sp. These findings underscore the substantial resilience of wild subtilisin in diverse organic solvents, a quality essential for industrial applications.

A correlated research study on a distinctive alkaline protease extracted from *B. megaterium*-TK1 illustrated the impact of several organic solvents on its effectiveness wherein methanol displayed 88% of relative activity (Manavalan et al. 2020). Given the multitude of reports on the impact of organic solvents on protease activity, it explains that each protease is distinctly influenced in its activity. In addition, methanol exhibited a relative activity of 84% when treated with extracellular serine protease from *Geobacillus* sp. GS53 which is lesser than the current study, while benzene increased the activity at ~ 95% (Baykara et al. 2021). Methanol, ethanol, and acetone’s presence rose the proteolytic activity more than 100%. However, with 2-propanol (20, 40, 80%), 9, 12, and 1% respective activities were decreased. Chihuil semi-purified proteases extract (SPE) high stability in organic solvents suggests its potential application in catalytic processes to improve nonpolar substrate solubility or prevent microbial contamination in media reactions (Rios-Herrera et al. 2020).

#### Wild subtilisin substrate specificity and kinetic features

Assessing interaction of substrateswith purified wild subtilisin involved exploring the relative activities (%) with various substrate forms, as presented in Table 2. Wild subtilisin shows the cleavage of both natural casein and modified azo-casein. Additionally, it exhibited esterase and amidase activities in ATEE and BTEE. Remarkable catalytic proficiency was observed as wild protein efficiently acted on N-Suc-F-A-A-F-*p*NAwith 98.7%.Empirical data supports the characterization of wild subtilisin, demonstrating its selectivity for substrates that are hydrophobic with names like N-Suc-F-A-A-F-*p*NA, wherein aromatic residues appear around P1 and P4 position. The amino acids with aromatic properties possess a crucial function in initiating the robust reaction that produces wild subtilisin at the location of catalysis.

According to prior literature, elevated efficiencies have been observed for peptides that are synthetic in material. For instance, a fibrinolytic enzyme AprE127 from *B. subtilis* purified through sequential chromatographies exhibited increased amidolytic activity, particularly with N-Suc-Ala-Ala-Pro-Phe-*p*NA (Frias et al. 2021). Our results correspond with other studies that also exhibited a strong selectivity for casein (substrate). For instance, thermostable protease exhibited comparable characteristics (Baykara et al. 2021).Wild subtilisin exhibited the ability to facilitate the breakdown of both unaltered and modified proteins, along with synthetic peptides. The presence of a few particular amino acids just preceding the bond between peptides that undergoes fragmentation determines wild subtilisin's specificity toward the substrate (Shettar et al. 2023b). Another study reported casein as the maximum effective hydrolytic substrate acting towards thermostable alkaline protease (Manavalan et al. 2020). Casein had been demonstrated to be the best substrate for proteolytic action of alkaline serine protease, with a 100% efficiency. Soy protein isolate, fish protein, as well as glycoproteins exhibited relative enzyme activity of 51%, 45%, and 33%, respectively. In comparison, ISP-SW5 had lesser relative effectiveness of the enzyme towards keratin and bovine serum albumin (BSA), respectively (Yang et al. 2020). The enzyme hydrolyzes practically all of the investigated substrates (gelatin, casein, skim milk, BSA, and bovine hemoglobin), making it an ideal detergent ingredient. The outcomes of specificity for substrates determination revealed casein as best substrate for prot DS5 (Wen et al. 2022).

The kinetic properties of the wild subtilisin had been scrutinized employing the Lineweaver–Burk plot, it also exhibited classical Michaelis–Menten kinetics and the results are detailed in Table 3. Subtilisin activity exhibited a gradual increase with escalating substrate concentration, eventually reaching a saturation point that signifies the active site's saturation.The K_m_ values for casein and N-Suc-F-A-A-F-*p*NA were assessed at 0.952 ± 0.8 and 0.731 ± 0.5 mM, respectively. Simultaneously, the V_max_ values were determined to be 1.157 ± 30 × 10^3^ and 0.87 ± 9 × 10^3^ U/mg for casein and N-Suc-F-A-A-F-*p*NA, correspondingly. A much lower K_m_ value suggests heightened affinity for substrates alongside robust adherence to the substrate. The K_m_ values suggest a stronger affinity of the subtilisin for the N-Suc-F-A-A-F-*p*NA substrate.The enzyme's V_max_ is the rate of activity that it proceeds until it approaches maximal saturation when interacting with the substrate. The determined k_cat_ and derived catalytic efficiency k_cat_/K_m_ for casein were 2.314 × 10^3^ min^−1^ and 2.43 × 10^3^ min^−1^ mM^−1^, respectively. Similarly, for N-Suc-F-A-A-F-*p*NA, the values were 1.74 × 10^3^ min^−1^ and 2.38 × 10^3^ min^−1^ mM^−1^, respectively as shown in Table 3.

**Table 3:** Kinetics of purified wild subtilisin

Substrate	*K*_*m*_ (mM)^a^	*V*_*max*_ (X10^3^ U/mg)^a^	*k*_*cat*_ (X10^3^ min^−1^)	*k*_*cat*_*/ K*_*m*_ (X10^3^ min^−1^ mM^−1^)
Casein	0.952 ± 0.8	1.157 ± 30	2.314	2.43
N-Suc-F-A-A-F-*p*NA	0.731 ± 0.5	0.87 ± 9	1.74	2.38

^a^Values denoted are mean ± standard deviation of three different enzyme assays

In a fascinating exploration involving natural fermentation, a reduction in K_m_ of two novel fibrinolytic enzyme JP-I and JP-II indicated an enhanced specificity of JP-I enzyme (Kim et al. 2020). The K_m_ and V_max_ of 0.197 mg/mL and 1.22 × 10^3^ U/mg, respectively, were observed for the purified protease. The lower K_m_ and higher V_max_ indicate stronger affinity of enzyme and superior catalytic efficiency for its substrates, and this coincides with the values obtained for wild subtilisin kinetic parameters (Tarek et al. 2023). The thermophilic and alkaliphilic protease exhibited V_max_ of 344.83 mg/mL/min and K_m_ vale of 100.04 mg, indicating the protein is a highly efficient with a considerable affinity that recognizes the casein substrate (Shad et al. 2024).

### Analytical characterization of purified wild subtilisin

#### Thermogravimetric analysis

**Fig. 5:**
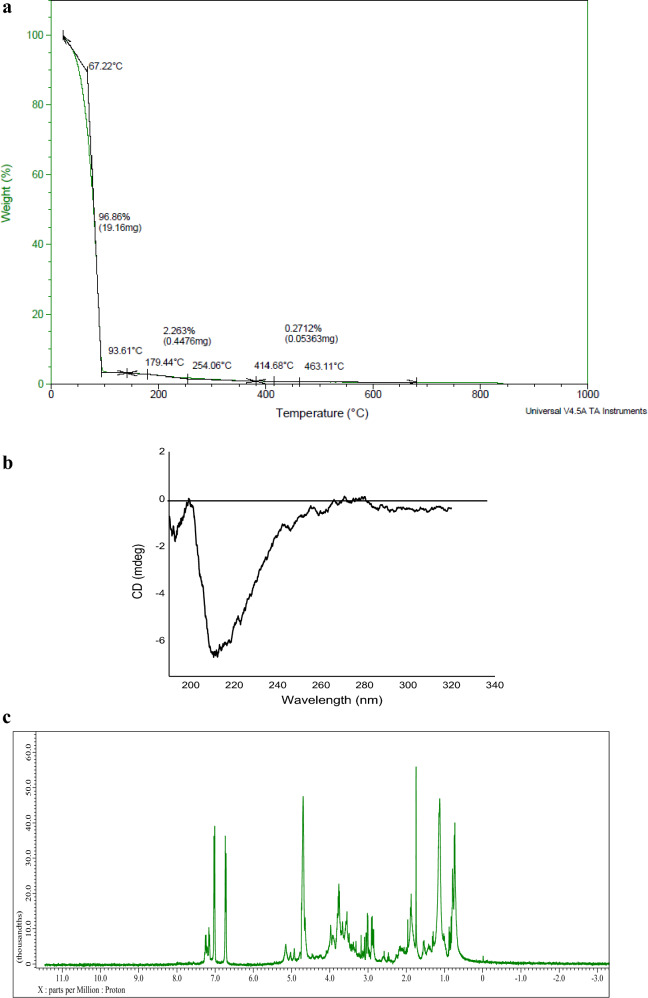
Characterization of wild subtilisin using bioanalytical techniques (**a**) TGA (**b**) CD (**c**) ^1^H-NMR

TGA is capable of offering beneficial details pertaining to their thermal resilience, and it also reveals the possibility of residual pollutants or molecules of organic in nature on their exteriors.The TGA thermogram illustrated in Fig. 5A depicts twomajor phases of mass reduction (%) at corresponding temperatures (°C).During the initial phase, there was a major weight loss of 96.86% observed between the temperature range of 67–93 °C, signifying the desiccation of physically adsorbed water on protein’s surface.The subsequent phase of mass reduction, involving a minor loss of 2.26%, took place within the temperature interval of 93–254 °C, further enhancing the desiccation process of the protein. Post 254 °C, the protein goes under complete decomposition with minor loss of 0.2%.Weight loss becomes noticeable once temperatures surpass 260 °C. As the temperature approaches 400 °C, the result is the generation of carbonaceous char Bagewadi et al. (2020). The TGA examination of wild subtilisin showcased the protein's remarkable thermal resilience. The protein's structural complexities, content, and configuration have each got an impact on its breakdown by temperature behavior.

The acquired results have a relationship to the existing TGA patterns published in the research that is available. Comparable observations regarding the thermal breakdown of raw sample of tannery fleshing were also documented by Kumar V et al. (2023). Boudreau et al. (2023) investigated the enzymatic exposure associated with employing protease and lipase to cleanse away any lingering meat from salmon frames to isolate collagen-containing hydroxyapatite (sHAP), which demonstrated that both the lipids and proteins that were not collagenous retrieved from the salmon set were subjected to thorough hydrolysis upon thermal analysis. Another researcher documented the thermal properties of BP1, a protein modified through recombinant technology, displaying degradation at higher temperatures (Tachibana et al. 2021). The thermal durability of cocoonase has been evaluated employing CD spectroscopy, and its thermal characteristic was assessed utilizing Differential Scanning Calorimetry (DSC) ultimately explaining its heating properties and stability (Rani et al. 2024).

#### Circular dichroism

CD spectroscopy provides valuable insights on the secondary arrangement and configuration of peptides and protein molecules, encompassing α-helix, β-sheet, and randomly assembled coil structures. The wavelength spectrum demonstrated negative ellipticity across the 200–230 nm region, featuring a high point at 211 nm, indicating the helix-like form found in wild subtilisin as indicated in Fig. 5B. In a phosphate-buffered solution, CD graph of the purified wild subtilisin protein displays negative peaks, strongly indicating the presence of an α-helical configuration. A favourable signal at 270 nm indicates the existence of disulfide bonds.Additionally β-sheet content was incorporated in protein. The expansive negative band spanning between 279 and 320 nm exposes existence of β-conformation.Proteins possessing α-helical variations are more inclined to transform into membrane-active molecules.

The CD spectra analysis of the serine protease inhibitor Kazal type 1 (SPINK1), as explored by (Buchholz et al. 2020), exhibited a negative point at the wavelength between 210 and 220 nm, indicating the presence of an α-helical content. The amide chromophore is responsible for the CD spectra observed in α-CHT as explained by (Akram et al. 2020). The assessment of secondary structure involves analyzing CD counts associated with amide—amide interactions. In a similar literature study, the negative peak spanning from 207 to 223 nm, along with a positive peak around 196 nm, indicates a robust presence of β-sheets and a reduction in random coil contents (Sharma et al. 2020). This study goes in line with the current work. Another study reported using far-UV circular dichroism (CD), it was discovered that serine protease's secondary framework demonstrated a slight transition within the molten globule (MG) condition intermediate, pH 3, compared with its original state, a pH value of which is characteristic for MG. UV-near CD revealed the greatest drops from 228 to 300 nm (Jamal et al. 2024). Cocoonase, a protease formed at the time the formation of silk moths, was gathered and refined, along with its secondary composition was investigated utilizing CD spectroscopy. The results demonstrated an abundance of α-helix 4.3%, β-sheet 55%, turn 8%, and random coil 32.7% (Rani et al. 2024).

#### ^1^H-nuclear magnetic resonance

The assessment of the structure of purified wild subtilisin was extended through the utilization of ^1^H-NMR, as illustrated in Fig. 5C. The absorption spectral peak in the range of 1–1.3 ppm indicates the occurrence of threonine (Tr) and methylene groups, whereas those at 3.2 ppm are associated with cystine (CH_2_–S–S), arginine (Arg). Peaks located at 1.5 ppm (minor), 4.0 ppm, long peak at 4.8 and 7.0 ppm were designated to alanine (Ala), serine (Ser), lysine (Lys), and tyrosine (Tyr), correspondingly (Sharma et al. 2022).The distinctive signals from the α-protons are evident in the δ range of 3.7–4.7 ppm (Shettar et al. 2023b). Thus, the ^1^H-NMR analysis of the liquid purified wild subtilisin confirmed the liberation of amino acids.

Similar peaks have been found in the literature studied. The number of peaks identified in the NMR of current study coincides with the work reported on keratinase from *B. velezensis* NCIM 5802 and confirmed the various amount of residues of amino acids in the hydrolysates (Sharma et al. 2022). In another scenario of α-aminophosphonic acids, the ^1^H-NMR spectra indicate the occurrence of distinctive chemical shifts for phenolic and theophenic protons, observed between 6.60 and 7.61 ppm (Tlidjane et al. 2022). A report on utilizing a crude protease to r extract fat from tannery fleshing wastes was subjected to NMR studies revealing similar peaks that revealed the occurrence of fatty acids that are unsaturated (Tujjohra et al. 2023). A recent work on enhancing the fibrinolytic properties of subtilisin NAT (nattokinase) revealed indications of unsaturated fatty acids in the spectra of ^1^H NMR and ^13^C NMR. The shift in chemical composition and strength of detected signals led to the identification of linoleic acid as a key component (Takagaki et al. 2020).

### Varied applications of wild subtilisin

#### Goat skin dehairing

**Fig. 6:**
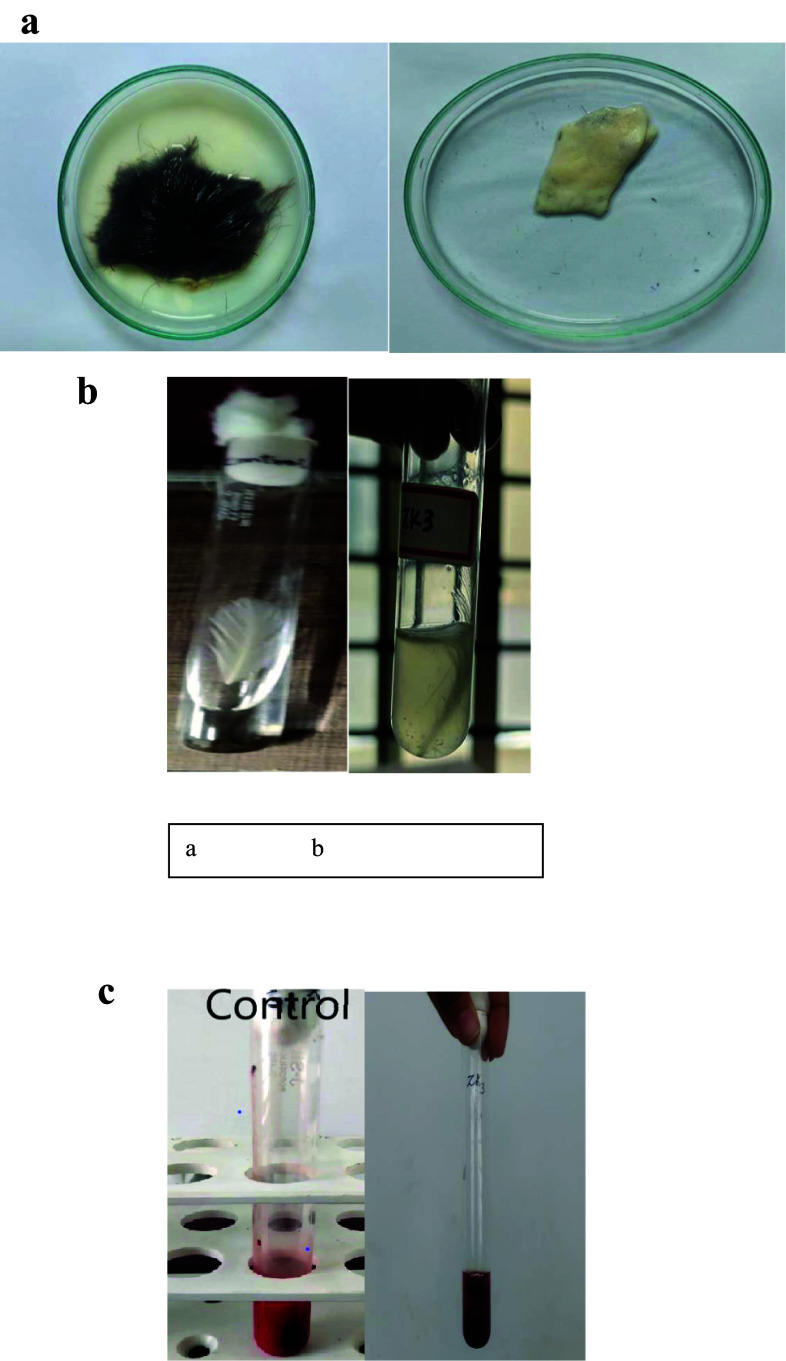
Applications of wild subtilisin (**a**) Goat skin dehairing (**b**) Feather degradation (**c**) Blood clot dissolution

Skin tissue pieces measuring 4 × 4 cm were immersed in wild subtilisin solution and incubated at a temperature of 40 ℃. As time progressed, there was an observable increase in the detachment of hair from the tissue. After 6 h, it appeared that the skin tissue had reached a point with the protein, coinciding with the moderate level of dehairing. Subsequently, at the 23 h, the external solution exhibited turbidity, indicating the degradation of certain skin proteins which facilitated the loosening of the connective tissue. The hair was completely detached at this time position (Fig. 6A).

(Nilegaonkar et al. 2007) documented that buffalo hide was successfully dehaired by 1% crude protease post a 21-h period at 28 ℃ and a pH of 7.0. Research on keratinolysis revealed that goat skin hair could be easily removed within 24 h of exposure to alkaline protease, as opposed to skin treated solely with a buffer (control) (Kandasamy et al. 2018). Matkawala et al. (2019) documented that in contrary to the control group, hairs were readily detached post 24 h of incubation when employing both enzymatically as well as chemically processed skin. This procedure displays importance in diminishing toxicity while concurrently improving leather quality through the utilization of biomolecules rather than conventional commercial chemicals. ZMS-2 extracellular serine protease's catalytic potentials have been demonstrated utilizing scanning electron microscopy using casein pellets and dehaired skin (Khan et al. 2022). Overnight treatment with partly purified protease resulted in hair removal of cow hide. Following the enzymatic administration, the skin hairs were taken out effortlessly using forceps. Unlike to the chemically processed cow skin, hairs appeared significantly simpler to eliminate post exposure with the protease (Manavalan et al. 2020). G3726 a novel serine protease expressed by *B. subtilis* strain SCK6 was able to completely dehair goatskin within 4 h without causing any damage, outperforming the chemical agent Na₂S (Li et al. 2022). The SptA protease effectively dehaired goatskins within 6 h of treatment without compromising the grain or collagen structure, positioning it as a promising candidate for leather production (Wang et al. 2023).

#### Blood clot dissolution

The investigation delved into the influence of the wild protein on the disruption of blood coagulation in vitro. The use of water in this assay failed to reveal any signs of blood clot dissociation, as shown in Fig. 6B(a). In a span of 6 h, wild subtilisin operating at 37 ℃, thoroughly dissolved the blood clot. The outcomes of this procedure are depictedin Fig. 6B(b). The protein proficiently solubilized the blood clots, and the dissolution exhibited a notable increase with time. In a time frame of 6 h, the wild subtilisin successfully dissolved 86.8% of the blood clots.

In a correlated investigation, a serine protease fibrinolytic enzyme termed ACase, derived from mushrooms, effectively dispersed blood clots, with the rate of dissolution escalating over time. In just 3 h, the enzyme successfully dissolved 82% of the clots from the blood (Li et al. 2021). The research, as reported by reference(Sharma et al. 2019), concluded that a fibrinolytic protease obtained from *B. cereus* RSA1 facilitated the complete solubilization of blood clots within 4 h at 37 ℃. An isolated protease from *Moringa oleifera* Lam. leaves demonstrated fibrin digestion as well as blood clot dissolution activity in vitro, indicating fibrinolytic ability in dissolving clots in the blood (Sawetaji and Aggarwal 2024). Alkaline protease, a fibrinolytic enzyme, represents the most effective candidate for treatment of cardiovascular disorders alongside breaking down blood clots. *Streptomyces violaceoruber* is a potent generator of alkaline protease, which dissolves blood clots in a timely way. Protease is widely regarded as the most effective treatment for blood clots caused by cattle slaughter as studied and reported by (Al-Dhabaan 2024). The study of proprotein convertase subtilisin/Kexin type 9 (PCSK9) responsible for cardio events revealed that individuals with diagnosed cardiovascular disease (CVD) and elevated PCSK9 levels faced a 52% greater risk of experiencing future cardiovascular events compared to those with lower PCSK9 levels. Elevated PCSK9 levels are strongly associated with a heightened risk of future cardiovascular events. These findings expand the understanding of PCSK9's role and highlight the potential clinical applications of PCSK9 inhibitors (Liu et al. 2021). The findings of a study suggested that the enzyme derived from *B. subtilis* C10 is likely a serine protease with significant fibrinolytic activity (Thu et al. 2020). Another enzyme AprEBS2 secreted by *B. velezensis* BS2 depicted strong α-fibrinogenase and moderate β-fibrinogenase activity (Yao et al. 2019).

#### Feather degradation

The investigation on feather disintegration indicated that the wild subtilisin effectively hydrolyzed a substantial amount of protein, demonstrating its efficiency in the extensive degradation of chicken feathers (Fig. 6C). This enzymatic capability holds promise for the potential mitigation of such waste, suggesting a prospective avenue for waste management.

In line with the current research, several researchers have worked on serine and alkaline cold active proteases which have shown excellent results for feather degradation (Mushtaq et al. 2021a, 2023). A study revealed the breakdown of autoclaved chicken feathers was noted in the abundance of purified alkaline serine protease post 24 h at 37 °C (Bhunia et al. 2013). The disintegration of whole chicken feathers by utilizing keratinolytic enzyme have displayed good results, similar to wild subtilisin (Fakhfakh-Zouari et al. 2010). Another work effectively purified cold-active serine alkaline protease, which may efficiently digest resistant solid waste materials like feathers resulting in various products with added value (Sengupta et al. 2023). A keratinolytic protease was produced from *B. subtilis* ES5 which showed potential keratinase properties of degrading the chicken feather in order to be used in several biotechnological applications (Alamnie et al. 2023).

### *Insilico* analysis

#### Functional and physiochemical analysis of subtilisin

**Fig. 7:**
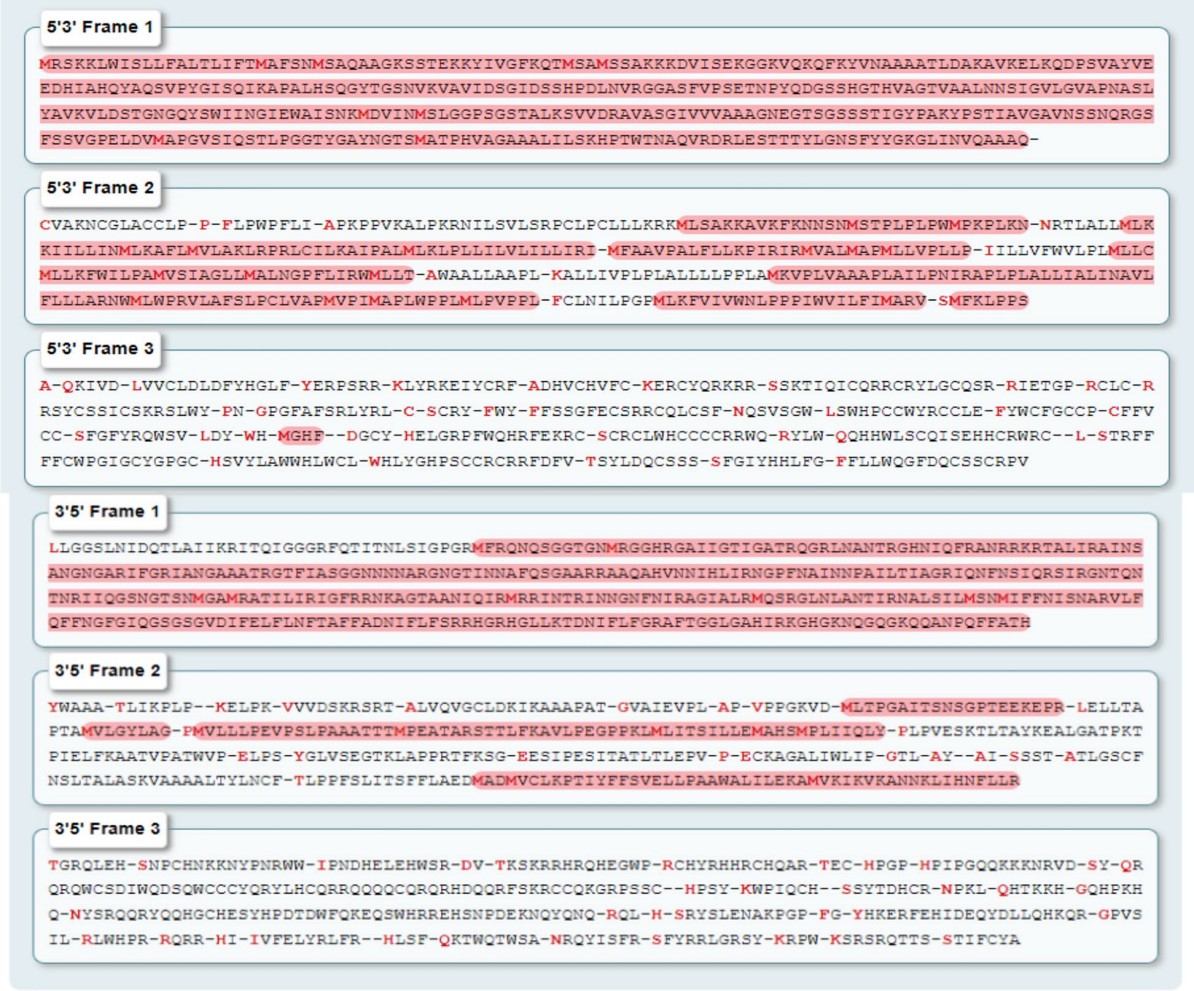
Translated protein sequence of subtilisin

The gene sequence obtained previously (Shettar et al. 2023b) from the GenBank accession number of *B. subtilis* strain ZK3 (Shettar et al. 2023a) was used to translate to protein sequence using the Expasy translate tool. The possible translated frames were obtained as shown in Fig. 7. The 5′3’ open reading frame which is completely highlighted in red color is the translated protein sequence of subtilisin. The MEGA 11 application has been applied for constructing the tree of phylogenetic relationships of the gene and protein sequence and is displayed in Fig S1 and S2 in SI, respectively. The closely related protein sequences of subtilisin from different *Bacillus* sp. is evidenced in the tree.To discover protein realms, relationships, and operational sites, utilize the ScanProsite analysis utility to look through the subtilisin sequence of proteins seeking matches towards PROSITE characteristics and trends.The functional prediction of the subtilisin protein sequence revealed the presence of a subtilase (peptidase S8) domain spanning amino acid positions 111–380 within the sequence. This domain encompasses the catalytic triad of subtilisin, consisting of ASP138, HIS170, and SER327 with a score of 60.853 (Fig S3 in SI). It also displays the ranges of active site residues of aspartic acid, histidine and serine.Employing a similar method termed InterPro Scan, a comprehensive sorting of sequences of proteins across the families was conducted, highlighting pertinent regions and preserved regions. Fig S4 in SI explains about the confirmatory details of subtilisin’s domain belonging to peptidase S8 family subtilisin-related. The catalytic triads at D138, H170 and S327 exactly coincide with the results from ScanProsite. The domain section has Subtilisin_Carlsberg-like, peptidase S8/S53 which represents that the subtilisin sequence form the current study consists of parts belonging to the mentioned domains and also is part of the homologous superfamily as shown in the figure. InterPro integrates all resources into a single entry, eliminating redundancies while retaining the essential details from each source in its output. The web platform provides a comprehensive array of data from its annotation results, including a distinctive name, accession ID, Gene Ontology descriptors, and entry categories such as family, domain, repeat, site, and homologous superfamily (Blum et al. 2021). By summarizing the results from both the tools, the results are same and depicts that subtilisin has a catalytic triad and belongs to peptidase S8 family. The physicochemical attributes were computed utilizing the ProtParam tools on the Expasywebsite. This analysis aimed to ascertain the amino acid count, molecular mass, theoretical isoelectric point (pI), GRAVY, instability, and lastly, the aliphatic index.The general molecular weight of the serine proteases varied within the range of 38–48 kDa (withsubtilisin exhibiting a molecular weight of ~ 39 kDa), nearly aligning with the in vitro observations of a band below ~ 45 kDa. The theoreticalpI value for the purified protein was 9.25 obtained from ProtParam tool. An instability index predicted to be < 40 suggests protein stability, whereas values exceeding 40 indicate protein instability (Guruprasad et al. 1990).The instability index of subtilisin exhibited a value of 30.63. This value signifies the stability of the serine protease.An elevated aliphatic index signifies the thermostability of a protein across a broad temperature range (Ikai 1980). The aliphatic index had a specific value of 82.23 for subtilisin, indicate enhanced thermo-stability over a wide temperature spectrum.Moreover, the biochemical analysis revealed that subtilisin maintains stability up to 60 ℃.The count of positively charged amino acids (Arg + Lys) = 30 exceeded that of amino acids that are negatively charged (Asp + Glu) = 23. The structural diversity of protein is influenced by the presence of both negatively and positively charged amino acids.A low GRAVY score indicates that the protein is minimal hydrophobic yet highly hydrophilic in nature meaning that it interacts well with water. The GRAVY index in this case is − 0.059.

The discovered strain of thermophilic and alkaliphilic protease exhibited 99.77% similarity to *Bacillus subtilis* K-3 via 16 s rRNA sequencing (Shad et al. 2024). According to sequence evaluation, ISP-SW5 an intracellular alkaline serine protease gene possesses a 960-bp open reading frame that codes for a protein with 319 amino acid sequences (Yang et al. 2020). The catalytic triad DHS is acknowledged as a crucial element in the catalytic process of serine proteases. A literature study reported the identification of the domain of alkaline serine protease (AKD9 gene) produced from *B. subtilis* D9, which corresponded to peptidase S8 (subtilase) family, which goes in line exactly with the current investigation. It also has specific serine protease catalytic triad (Raj et al. 2017; Mahmoud et al. 2021).The AKD9 gene has also resemblances with subtilisin as it has shown similar results for the instability index to be < 40, also proves to be thermostable and hydrophilic protease. Another study reported on novel keratinase from *B. cereus* classifies itself as a member of peptidase S8 family and the gene revealed same catalytic triad of serine protease proving it to be subtilisin-like serine protease, just like subtilisin from the current research work. The physiochemical parameters reveals it to be thermostable (Bhatt and Singh 2020; Almahasheer et al. 2022). ISP-SW5 is closely linked to intracellular serine proteases from the peptidase S8 family, as revealed by phylogenetic study. Based on the developmental route, the examined strains HR01 and HR02 exhibited a significant inclination for *B. subtilis* cd4 and *Bacillus sonorensis* SRCM101395, respectively which produced robust thermostable protease (Homaei and Izadpanah Qeshmi 2022). The findings of cold-active alkaline serine protease (CAASPR) revealed a tight relationship regarding the serine peptidase family (S8A). Although 48 kDa was its molecular weight, calculated theoretical isoelectric point (pI) stood at approximately 5.98 (Mushtaq et al. 2023). The serine protease's amino-acid composition suggests that belongs to the serine protease enzyme that consists of D, H, S in the catalytic site. Bioinformatic research with Protparam revealed that the enzyme had a value pI of 6.02, low instability index, strong aliphatic index, and a small GRAVY value (Syahbanu et al. 2020).

#### Homology modelling and molecular docking

**Fig. 8:**
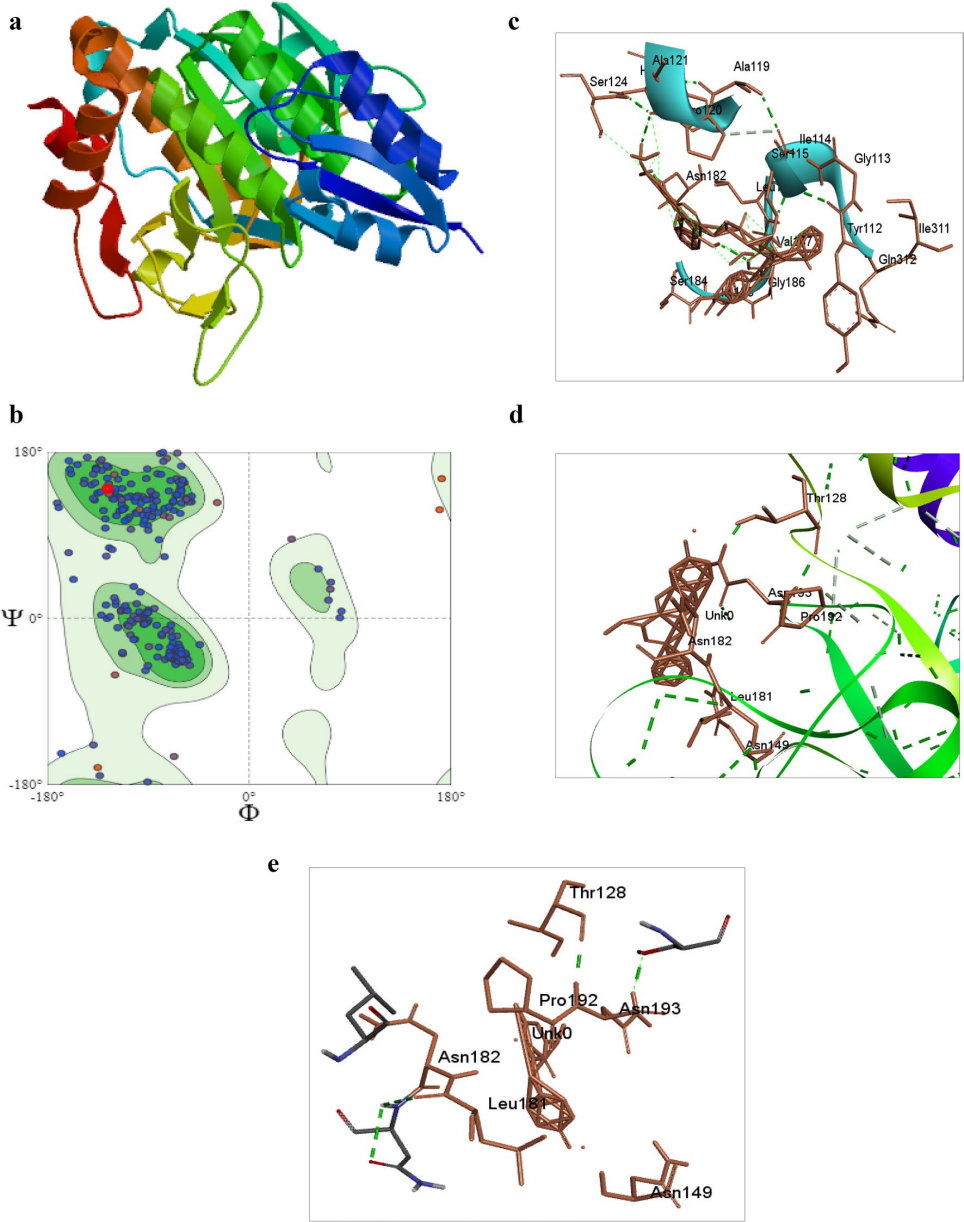
Homology Modelling and Molecular docking (**a**) 3D structure of template (PDB: 3WHI) (**b**) Ramachandran plot (**c**) Molecular Docking ofSuc-Phe-Ala-Ala-Phe-*p*Nawith subtilisin (**d**) Molecular Docking ofN-Benzoyl-L-tyrosine ethyl ester (BTEE) with subtilisin (**E**) Molecular Docking ofN-Acetyl-L-tyrosine ethyl ester monohydrate (ATEE) with subtilisin

For a thorough comprehension of the mechanisms employed by the subtilisin enzyme under heightened pH and temperature conditions, coupled with remarkable substrate specificity, a homology modeling approach was employed via SWISS-MODEL. As of now, the specific structure of subtilisin remains undiscovered, but it is hypothesized to bear resemblance to numerous subtilisins, especially those exhibiting higher sequence identity and whose structural forms have been found. Thus, the translated protein sequence for subtilisin has been utilized as input to generate a preliminary version applying SWISS-MODEL. A comprehensive model was developed and the existing 3D structure of unautoprocessed form of IS-1 inserted pro-subtilisin E (PDB ID: 3WHI), showcasing a 94.03% sequence identity with the protein sequence of subtilisin, was employed as a template (Fig. 8A). The visual representation of the template was displayed in a cartoon format using PyMol software.A Ramachandran plot ended up being utilized in order to assess the stereochemical durability associated with the subtilisin molecule and give insights into its structural properties. In Fig. 8B, 93.04% of the amino acids had been observed in the most favored region, while only 0.87% of the remaining residues located inside outlier region. This distribution suggests the model in question possesses high accuracy and equilibrium, making it suitable to conduct in silico trials. The MolProbity score is 2.37. The predicted model's quality factor was determined to be 90.71 using ERRAT2 (Fig S5 A and B in SI). The evaluation with VERIFY3D further endorsed the positive quality of the model, as a minimum of 80% residues must score ≥ 0.2 in 3D/1D profile. In this particular case, a value which is equivalent to or larger than 0.2 was achieved by 89.34% of the residues, confirming the overall quality of the model (Fig S6 in SI) via SAVES v 6.0 tool. The variability of proteins, especially in their highly flexible loop regions, plays a vital role in enzyme catalysis. However, these loops often become vulnerable to heat and are typically the first to unfold under extreme conditions. In this study, FoldUnfold tool was utilized to predict regions of high flexibility and disorder within the subtilisin sequence. The tool identified 8 unfolded regions spanning the 381 aa subtilisin sequence. The 8 regions comprised of different lengths of amino acids (aa) namely, length 12 aa (26–37aa), length 19 aa (49–67aa), length 14 aa (78–91aa), length 21aa (154–174)aa, length 12 aa (231–242aa), length 16 (259–274aa), length 17 aa (283–299) and length 13 aa (311–323aa) as shown the Fig S7 in SI. A study reported identification of highly flexible disordered segments of keratinase KerBpusing FoldUnfold (Su et al. 2022).

Further, molecular docking of 3WHI protein molecule was carried with all 3 substrates (Table S1 in SI). The prepared ligands and protein were subjected to docking, while the specifics of binding energy are compiled in the Table 4 and Fig. 8C, D, Ecorresponding to N-Suc-F-A-A-F-*p*NA, BTEE and ATEE respectively.The outcomes of docking explained that subtilisin attained the highest binding affinity of − 8.8 kcal/mol with N-Suc-F-A-A-F-*p*NA, and details of the amino acid relations are provided in the Table 4 and Fig. 8C using Discovery Studio software, exhibiting excellent binding affinity. Additionally, as there were rotatable bonds, flexible docking and clustered docking poses were employed to prioritize conformations with consistent interactions with subtilisin and validated docking poses through MD simulations. The higher number of rotatable bonds in ligands can influence the accuracy of molecular docking by (i) increasing the conformational search space, making it challenging to identify the most relevant binding pose, (ii) introduce an entropic penalty upon binding that may not be fully captured in docking scores, (iii) lead to multiple low-energy binding poses, reducing result reliability, and (iv) potentially cause overestimation of binding affinity due to improper sampling of ligand conformations. However, in current study, ligand flexibility was carefully evaluated and ensured that docking parameters were optimized to mitigate this effect (Alhumaid and Tawfik 2024). The amino acids involved in molecular docking interactions are PRO120, SER124, GLY186 with distances of 2.62 Å, 4.0 Å, 3.69 Å. Similarly, Fig. 8D and E displays the docking of BTEE and ATEE with subtilisin protein showing − 7.6 kcal/mol and − 6.0 kcal/mol binding energy respectively along with interacting residues. The findings from molecular docking analyses highlight the participation of several amino acid residues in the interaction between subtilisin and various substrates. Notably, residues such as THR 128, LEU 181, ASN 149, PRO 192, ASN 193 and ASN 182 appear in the docking results across the substrates. Consequently, it is inferred that these amino acids contribute to forming the active site of the subtilisin enzyme. This enables substrate interaction and binding to the protein, supporting the catalytic process.

**Table 4:** Molecular docking results of 3 substrates with subtilisin protein

Ligand	Binding energy (kcal/mol)	Hydrogen interacting residue	Hydrophobic interactions
Suc-Phe-Ala-Ala-Phe-*p*Na	− 8.8	PRO120, SER124, GLY186	ILE114, VAL187, LEU188
N-Benzoyl-L-tyrosine ethyl ester (BTEE)	− 7.6		ASN149, LEU181
N-Acetyl-L-tyrosine ethyl ester monohydrate (ATEE)	− 6.0	ASN182	LEU181

*MDS* Molecular Dynamic Simulation

To delve into the biochemical characteristics more profoundly and investigate the relationships between structure and function via homology modelling, structural models for the SAPRH and SAPB enzymes were constructed, in a similar study. This involved utilizing the crystalline configuration of subtilisin NAT (PDB ID: 3VYV) as a reference template for the modeling process (Rekik et al. 2019). SOPMA and Swiss Model were employed to study 2D and 3D structures of W10 protein. The study found that it possessed 33.93% α-helices, 40.4% random coils, 15.85% β-strands, and 9.82% β-turns. It’s 3D structure ranked the most comparable (40.05%) to the template 3afg.2.A. subtilisin-like serine protease (Ji et al. 2020). In another similar study, the accessible three-dimensional structure of pro-subtilisin E (PDB ID: 3WHI), sharing an identity of the sequence of 65% with Pro-SAPN, served as the foundation for constructing the three-dimensional model of Pro-SAPN. The favored regions of the Ramachandran plot exhibited 93% of amino acids (Mechri et al. 2021). The I-TASSER service created a homology model for ISP-SW5 using a template of *B. clausii*'s intracellular subtilisin protease (PDB ID: 2 × 8j.1.A), that showed 54.64% similarity with ISP-SW5. I-TASSER's 3D structure reveals that Asp50, His87, and Ser246 in sticks form a catalytic triad (Yang et al. 2020). Nattokinase from *Bacillus subtilis* natto was employed as a template from Modeller v9.11, while 3D structure was acquired via the Protein Data Bank (Mushtaq et al. 2021b). Corresponding to the current work, the 3D structure of the protease from *Bacillus* sp. was modeled using Modeler 9v9. Subsequently, the generated configuration underwent evaluation for geometric errors and stability of energy through assessments utilizing RAMPAGE, VERIFY 3D, ERRAT, and PROSA and the final model which revealed to be reliable (Kandasamy et al. 2018).

The molecular docking score of − 7.108, characterized by an extra level of precision, delineates the favorable binding between lactose-CAASPR-HM49 and the subtilisin-like protease. A negative docking score signifies interactions between the two entities, suggesting favorable binding or affinity in molecular docking studies. Indeed, a total of seven hydrogen bonds were observed within the docked complex of lactose and the subtilisin-like protease from *Bacillus* sp. HM49 (Mushtaq et al. 2023). The docking studies of KerS13uv + ems and KerS26uv obtained from *KerS* gene of the mutated strains S13 and S26 utilizing the substrate N-succinyl-L-alanyl-L-alanyl-L-prolyl-L-phenylalanine4-nitroanilide, unveiled the influence of substitutions on the superimposed framework, indicating an enhanced binding affinity which implies to hold promise for applications in the industrial processing of feathers (Almahasheer et al. 2022). Another study explained the docking and MDS of Iturin A (from *Bacillus aryabhattai*) with Exo (1,3)-β-D-glucan synthase (from *Candida albicans*) revealed a significant level of affinity and stability, particularly with the active residues (Yaraguppi et al. 2021). Docking studies show that Subtilisin K2 and its fibrin substrate have a strong interaction, with negative ΔG (high binding affinity), indicating a complex binding pattern alongside association with essential residues for enzyme activity (Syahbanu et al. 2020). A researcher conducted blind molecular docking of the synthetic substrate N-succinyl-L-Phe-L-Ala-L-Ala-L-Phe-*p*-nitroanilide into the active site of a modeled serine protease from *Streptomyces mutabilis* strain TN-X30. The study identified the involvement of 21 amino acid residues in substrate binding, with a binding energy of − 4.3174 kcal/mol. Among these, the serine residue of the catalytic triad was highlighted as the key residue responsible for substrate catalysis (Mechri et al. 2022).

**Fig. 9:**
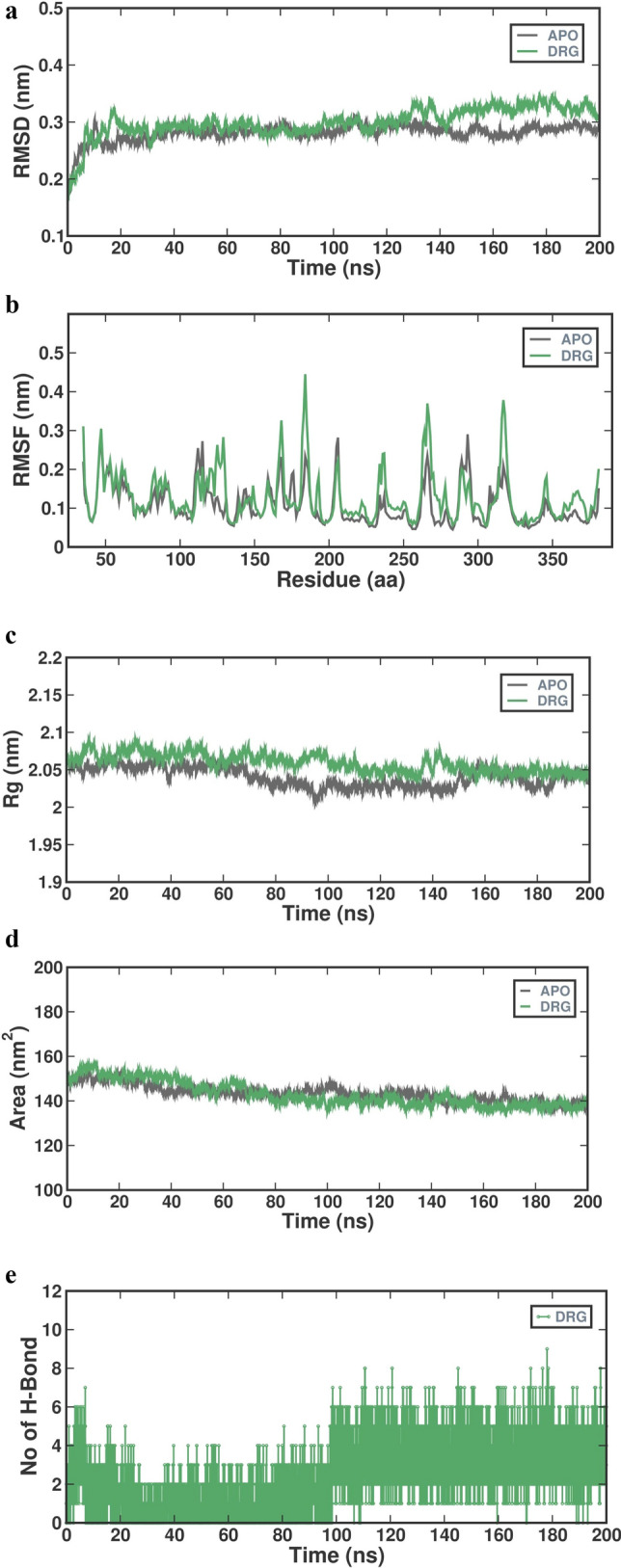
MDS analysisof APO and DRG complex (**a**) RMSD (**b**) RMSF (**c**) Rg (**d**) SASA (**e**) Intermolecular hydrogen bonds

Molecular dynamics simulations are an effective method for computing forces and analyzing the behavior of amino acid molecules.The robustness of the complex is examined by taking into account various parameters, including RMSD (Complex-Ligand), RMSF, SASA, Rg, and the examination of interactions of the hydrogen bonds.The ligand topology file for the protein–ligand complex is generated through the PRODRG server (https://davapc1.bioch.dundeeac.uk/cgi-bin/prodrg/submit.html). The protein's PDB file, containing the APO (subtilisin) state and the protein in conjunction with ligandsi.e. DRG (subtilisin, N-Suc-F-A-A-F-*p*NA), is uploaded to the PRODRG server and is named the same in all figures. Following this, the server generates an energy-minimized topology file, which is then utilized as input for simulations conducted with Gromacs.The RMSD figure was generated to demonstrate the coherent nature regarding the equilibrium among attributed and free subtilisin. Figure 9 Aillustrates an RMSD figure for the two different versions, APO and the Protein–Ligand connections of subtilisin. In the course of the first one to fifteen ns, there is a noticeable fluctuation, followed by the development of a steady arrangement that includes both ligand-associated as well as ligand-dissociated combinations right through the simulation.The average RMSD values for the APO protein, subtilisin in complex with N-Suc-F-A-A-F-*p*NA over the 200 ns duration are observed to be 0.264 ± 0.32 nm and 0.295 ± 0.13 nm, respectively.The estimated RMSD of the substrate (N-Suc-F-A-A-F-*p*NA) alone was approximately 0.21 ± 0.05 nm, indicating that the ligand maintained a stable conformation within the binding pocket throughout the 200 ns simulation. This is further supported by the minor increase in RMSD upon ligand binding and the overall system’s stability post-15 ns convergence.The RMSD figure demonstrated the comparatively stable state of the APO and Protein–Ligand complexities inside the entire system over completely 200 ns computer simulation. The RMSD convergence for both the protein and ligand occurred after 15 ns, and thereafter, remained consistently stable.Analysis of the ligand's interaction with hydrogen bonding and hydrophobic interactions, shows stable and persistent binding interactions throughout the simulation. These stable interactions further corroborate the ligand's binding stability. The homogeneity of the residues of amino acids inside of the protein's structure is evaluated utilizing RMSF, which also computes the protein's residue-wise divergence across the whole simulation. Higher RMSF values indicate increased flexibility and consistency of the residue, particularly noticeable with elevated RMSF numbers whereas a lower RMSF value indicates a stable enzyme—substrate complex.The RMSF graph illustrating the fluctuations of amino acid residues in the APO and complex [subtilisin, N-Suc-F-A-A-F-*p*NA] is presented in Fig. 9B. In the RMSF plot it can be seen that both APO and docked complex have shown fluctuations and also the docked complex showed higher RMSF value upto 0.45 nm and 0.29 nm for APO protein. The plot for protein–ligand complexexhibits higher RMSF in numerous regions, akin to APO, with minimal deviations from the APO. Therefore, the N-Suc-F-A-A-F-*p*NA molecule displays increased RMSF in the protein complex, indicating its greater stability and consistent equilibration with subtilisin throughout the 200 ns simulation. The Radius of Gyration (Rg) values offer insights into the protein's structural rigidity and compactness interior to the molecular dynamics environment during its interaction with the ligand. Throughout the 200 ns simulation duration, average Rg values for APO and the protein inside the complex along with N-Suc-F-A-A-F-*p*NA consistently fall within the range of 2.035 ± 0.010 nm and 2.075 ± 0.019 nm, correspondingly, as depicted in Fig. 9C.The structural complex of N-Suc-F-A-A-F-*p*NA with subtilisin exhibits heightened compactness, leading to improved stability. Throughout the simulation, N-Suc-F-A-A-F-*p*NA'sRg value remained largely constant, demonstrating its persistent compression and sturdiness over the whole 200 ns journey. Enzyme folding reflects the stability of the radius of gyration (Rg), whereas fluctuations in Rg signify the enzymes' unfolding. The assessment of SASA is performed to understand the regulation of the protein's function the interior of the system. The SASA for the complexes maintained constant and equilibrated throughout the simulation, demonstrating continuous stability for the entire 200 ns duration. The SASA values for subtilisin-APO and subtilisin with N-Suc-F-A-A-F-*p*NA are observed to be approximately 145.5 ± 5.12 nm^2^ and 148.5 ± 6.23 nm^2^, respectively. Assessing the alteration in SASA provides insights into the level of aggregation of complex proteins within the system. The SASA plot for subtilisin-APO andN-Suc-F-A-A-F-*p*NAis illustrated in Fig. 9D.The examination of hydrogen bonds in the protein–ligand complex, specifically with subtilisin, indicates a sustained binding of ligand to the protein subtilisin over the entire simulation period.Over the 200 ns duration of dynamic simulation, an average of 3 stable hydrogen bond pairs are observed for N-Suc-F-A-A-F-*p*NA. The continuing abundance of bonds made of hydrogen across the 200 ns computer simulation length demonstrates that N-Suc-F-A-A-F-*p*NA in complex with subtilisin, maintained its fundamental structure and balance. The formation of hydrogen bonds during molecular dynamic simulations are displayed in Fig. 9E. The graphs of RMSD, RMSF, Rg and SASA collectively demonstrate that N-Suc-F-A-A-F-*p*NA exhibits robust binding with subtilisin at its active site and maintains stability throughout entire simulation.

The RMSD of α-carbons among the original and improved simulation of subtilisin SAPN from *M. thermohalophilus* Nari2A^T^ equaled 0.85 Å, indicating significant resemblance between the overlaid model and the template. It represents the confirmation of the predicted framework for its application in future investigations (Mechri et al. 2021). A related research on subtilisin was used to predict the 3D structure using homology modelling. Superimposing the query's 3D structure on its template resulted in a combined RMSD values of 0.208 Å and Q-score of 0.966 (Mushtaq et al. 2021b). In another similar study of serine alkaline protease SAPRH, 2D-RMSD structure, depicting the RMSD revealed that the space of conformations explored due to the highest identified similarity sequence SAPB (from *B. pumilus* CBS) in the simulations was more extensive than that of the SAPRH model. Furthermore, as illustrated in the Rg, Van der Waals volume, and the potential energy U(r) of the atomic system, the values exhibit greater stability in the case of SAPRH compared to SAPB at temperatures of 343 K and 373 K, respectively (Rekik et al. 2019). Similar research on a biosurfactant Iturin A obtained from *B. aryabhattai* revealed that the RMSD convergence for both the protein (Iturin A) and ligand (Exo(1,3)-*β*-D-glucansynthase) occurred after 5 ns, and thereafter, remained consistently stable, exhibiting deviations within the range of 0.5–1 Å (Yaraguppi et al. 2021). Molecular dynamics (MD) simulation of thermostable serine protease revealed that, while the thermal endurance of this particular enzyme at 90° has been verified in the RMSD, RMSF, Rg, and SASA graphs, a pH level of 3 might profoundly alter the tertiary as well as secondary networks (Jamal et al. 2024). Similar to the present study, molecular dynamics simulations (MDS) was conducted on a modeled U32 collagenase. The study observed minimal deviations between the RMSD values of apocollagenase and the collagenase-collagen complex in the presence of the metal ion. It was concluded that smaller deviations indicate higher spatial equivalence between the initial and simulated states of the proteins, reflecting greater structural stability of the protein (Bhattacharya et al. 2019). The RMSD values for the study of nucleoside diphosphate kinase (NDK) protein with different substrates varied until 50 ns, after which they remained steady throughout the remainder of the simulation, stabilizing within the range of approximately 0.2–0.3 nm. This consistent RMSD reflects the stable configuration of the ligand under dynamic conditions in the system. The initial deviation observed up to 30 ns represents the extent of conformational adjustments in the protein–ligand complex during the molecular dynamics simulation. A lower RMSD value (in nm) signifies a more stable and favorable interaction between the ligand and the target protein (Bagewadi et al. 2023). The current investigation provides a glimpse into potential subtilisin through a comprehensive review of literature and characterization. Such insights can guide researchers in wet-lab studies aimed at identifying and characterizing the diverse range of serine and alkaline proteases or subtilisins produced by *B. subtilis.* Leveraging its untapped potential, *B. subtilis* emerges as a promising candidate in textile and leather industries and pharmaceutical biotechnology, offering numerous opportunities for exploration across various applications. Nevertheless, it is noteworthy that this protein has been relatively underexplored and infrequently reported in the existing body of research.

#### MMBPSA

The MMPBSA assessment is implemented to examine the attachment potency of subtilisin to N-Suc-F-A-A-F-*p*NA. The application of MMPBSA examines the subsequent processing of anchored complexes and the quantification of variances after the simulation. The ligand affinity is evaluated within the protein binding core where interactions take place. The stability of the protein–ligand complex largely depends on the binding energy, with negative values indicating greater stability.The findings suggest significant total binding energy, resulting in a value of − 118.623 ± 29.169 kJ/mol. It is noteworthy to highlight that the interaction between subtilisin and synthetic substrate N-Suc-F-A-A-F-*p*NA involves a substantial surface area. This strongly supports the anticipated intrinsic proteolytic activity, as previously mentioned in biochemical characterization section.

A research work reported binding energies in MMPBSA analysis by comparing the PCSK9 (proprotein convertase subtilisin/kexin type 9)prodomain/β-propeller interactions with wild-type and Asp374Tyr display similar binding energy of − 143 kJ/mol and − 165.5 kJ/mol respectively (Martin et al. 2020).Molecular dynamics (MD) simulations coupled with MMPBSA analysis of nucleoside diphosphate kinase (NDK) protein revealed that the total binding energy of the system was calculated to be − 153.802 ± 32.551 kJ/mol, indicating that the ligand remained consistently bound to the protein throughout the simulation. The system's total van der Waals energy was determined to be − 221.326 ± 21.020 kJ/mol (Bagewadi et al. 2023). The MMPBSA calculations were studied to identify the binding energies of subtilisin A with different cardiovascular related receptors which target infections and inflammation and among them, subtilisin A with C-reactive protein (CRP) showed − 70.71 kcal/mol determining the potential to modulate receptor activity (Dermawan and Alotaiq 2024). The binding free energies (ΔG_binding_) of subtilisin Carlsberg with carbon nanotube in different media were calculated as − 58.1 kcal/mol in aqueous media, − 46.3 kcal/mol in acetonitrile, and − 30.7 kcal/mol in heptane, aligning with the number of adsorbed atoms and the contact area. An analysis of the energy components indicates that van der Waals interactions are the primary driving force behind the binding (Zhang et al. 2016).

## Conclusion

In summary, the comprehensive study significantly advances the fundamental aspects of wild subtilisin and highlights its potential impact across diverse biotechnological applications. The successful multistage purification process, revealing a purified protein with enhanced activity and purification fold of 3.406, underscores its significance. Wild subtilisin was assessed with various factors of biochemical characterization to evaluate its activity and stability. The enzyme's versatility in applications such as depilation of goat skin, feather degradation, and blood clot dissolution demonstrates its wide-ranging industrial and healthcare potential. Additionally, the integration of advanced bioanalytical techniques like TGA, CD and ^1^H-NMR has provided enriched findings into structural and functional aspects of the wild subtilisin. The use of in silico approaches, including various tools, homology modeling, molecular docking, and dynamic simulations, has deepened the understanding of the enzyme's behavior with the specific substrate at the molecular level, offering valuable recommendations for future studies and applications. This synergistic approach of combining experimental and computational methodologies not only enriches the comprehension of subtilisin but also paves the way for its strategic utilization in both industry and research.

## Supplementary Information

Additional file 1.

## Data Availability

All data generated or analyzed during this study are included in this published article (and its supplementary information files).

## Abbreviations

SDS-PAGE: Sodium dodecyl sulfate polyacrylamide gel electrophoresis
SPR: Surface plasmon resonance
DSC: Differential scanning calorimetry
TGA: Thermogravimetric analysis
AFM: Atomic force microscopy
CD: Circular dichroism
^1^H-NMR: Nuclear magnetic resonance
DEAE FF: Diethyl aminoethyl fast flow
TCA: Trichloroacetic acid
PMSF: Phenylmethylsulfonyl fluoride
EDTA: Ethylenediaminetetraacetic acid
ATEE: N-acetyl-L-tyrosine ethyl ester monohydrate
BTEE: N-Benzoyl-L-tyrosine ethyl ester
*p*-NA: Para-nitroanilide
K_m_: Michaelis–Menten constant
V_max_: Maximum velocity
k_cat_: Turnover number
rRNA: Ribosomal ribonucleic acid
NCBI: National center for biotechnology information
BLAST: Basic local alignment search tool
MEGA: Molecular evolutionary genetics analysis
EMBL-EBI: European molecular biology laboratory-european bioinformatics institute
PLIP: Protein–ligand interaction profiler
GRAVY: Grand average of hydropathicity
MDS: Molecular dynamics simulations
RMSD: Root mean square deviation
Rg: Radius of gyration
SASA: Solvent accessible surface area
MMPBSA: Molecular mechanics poison surface area
