# Correction: Comprehensive biochemical, molecular and structural characterization of subtilisin with fibrinolytic potential in bioprocessing

**DOI:** 10.1186/s40643-026-01048-x

**Published:** 2026-05-06

**Authors:** Shreya S. Shettar, Zabin K. Bagewadi, Mohammed Alasmary, Basheerahmed Abdulaziz Mannasaheb, Ibrahim Ahmed Shaikh, Aejaz Abdullatif Khan

**Affiliations:** 1 Department of Biotechnology, KLE Technological University, Vidyanagar, Hubballi, Karnataka 580031 India; 2 Department of Medicine, College of Medicine, Najran University, 66462 Najran, Saudi Arabia; 3 Department of Pharmacy Practice, College of Pharmacy, AlMaarefa University, P.O. Box 71666, 11597 Riyadh, Saudi Arabia; 4 Department of Pharmacology, College of Pharmacy, Najran University, 66462 Najran, Saudi Arabia; 5 Department of General Science, Ibn Sina National College for Medical Studies, 21418 Jeddah, Saudi Arabia

**Correction to: Bioresources and Bioprocessing (2025) 12:21** 10.1186/s40643-025-00860-1

In this article (Shettar et al. 2025), The coordinates (x =  − 45.913, y = 51.057, z = 1.431) correspond to the catalytic active site of the enzyme. These coordinates were obtained using Discovery Studio (version 4.5). The original article has been corrected.

## References

[CR1] Shettar SS, Bagewadi ZK, Alasmary M et al (2025) Comprehensive biochemical, molecular and structural characterization of subtilisin with fibrinolytic potential in bioprocessing. Bioresour Bioprocess 12:21 40117024 10.1186/s40643-025-00860-1 PMC11928348

