# Supplementary material for: Comprehensive biochemical, molecular and structural characterization of subtilisin with fibrinolytic potential in bioprocessing
Source: Bioresour Bioprocess. 2025 Mar 21;12(1):21. doi: 10.1186/s40643-025-00860-1 (PMC11928348; doi:10.1186/s40643-025-00860-1)
Supplement: Supplementary file 1 — Additional file 1. [file 40643_2025_860_MOESM1_ESM.docx]

**SUPPLEMENTARY INFORMATION (SI)**

### Comprehensive biochemical, molecular and structural characterization of subtilisin with fibrinolytic potential in bioprocessing

### Shreya S. Shettar^1^, Zabin K. Bagewadi*^1^, Mohammed Alasmary^2^, Basheerahmed Abdulaziz Mannasaheb^3^, Ibrahim Ahmed Shaikh^4^, Aejaz Abdullatif Khan^5^

^1^Department of Biotechnology, KLE Technological University, Hubballi, Karnataka 580031, India, [01fe21rbt003@kletech.ac.in](mailto:01fe21rbt003@kletech.ac.in) (S. S. Shettar),[zabin@kletech.ac.in](mailto:zabin@kletech.ac.in) (Z. K. Bagewadi)

^2^Department of Medicine, College of Medicine, Najran University, Najran 66462, Saudi Arabia. [myalasmary@nu.edu.sa](mailto:myalasmary@nu.edu.sa)(M. Alasmary)

^3^Department of Pharmacy Practice, College of Pharmacy, AlMaarefa University, P.O. Box 71666, Riyadh 11597, Saudi Arabia. [bmannasaheb@um.edu.sa](mailto:bmannasaheb@um.edu.sa) (B. A. Mannasaheb)

^4^Department of Pharmacology, College of Pharmacy, Najran University, Najran 66462, Saudi Arabia. [iashikh@nu.edu.sa](mailto:iashikh@nu.edu.sa)(I. A. Shaikh)

^5^Department of General Science, Ibn Sina National College for Medical Studies, Jeddah 21418, Saudi Arabia.[aejaz@ibnsina.edu.sa](mailto:aejaz@ibnsina.edu.sa)(A. A. Khan)

Running title

**Purification and Characterization of Subtilisin**

^*^Correspondence to: Dr.Zabin K. Bagewadi

Associate Professor,

Department of Biotechnology,

KLE Technological University,

Vidyanagar, Hubballi – 580 031, India

Tel.: +91-0836-2378231; Fax: +91-0836-2374985.

Email: [zabin@kletech.ac.in](mailto:zabin@kletech.ac.in)

**Supporting Information: 5 pages, 1 table,7figures**

**Table S1**Molecular 2D and 3D structures of substrates

| Substrate Name | Pubchem ID | 2D structure | 3D structure |
| --- | --- | --- | --- |
| Suc-Phe-Ala-Ala-Phe-*p*Na  N-Acetyl-L-tyrosine ethyl ester monohydrate (ATEE)  N-Benzoyl-L-tyrosine ethyl ester (BTEE) | 99647946  2723594  77033 | 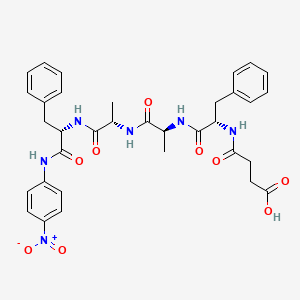  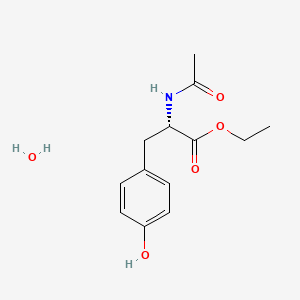  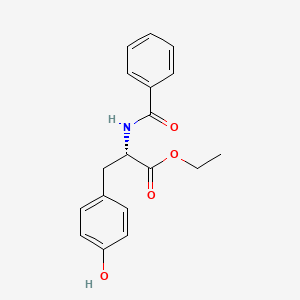 | 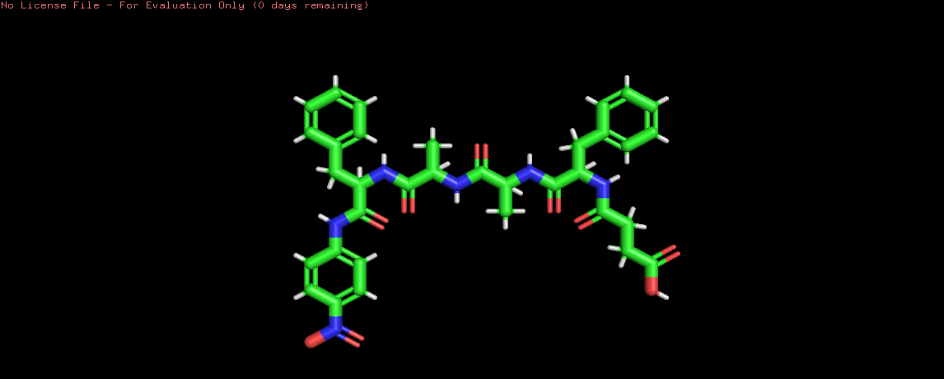  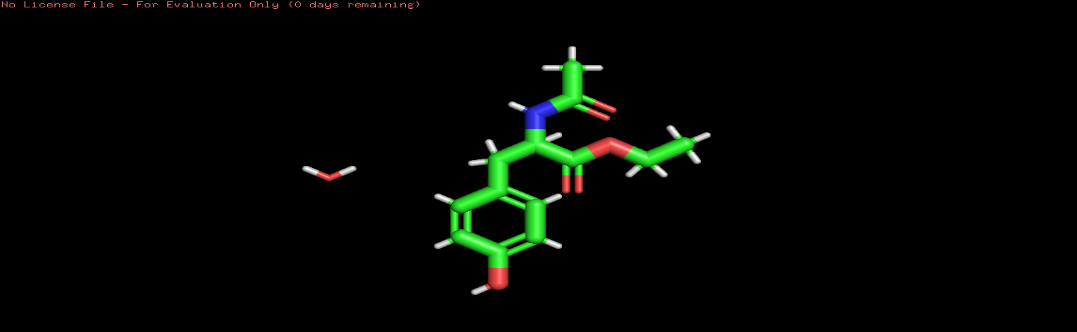  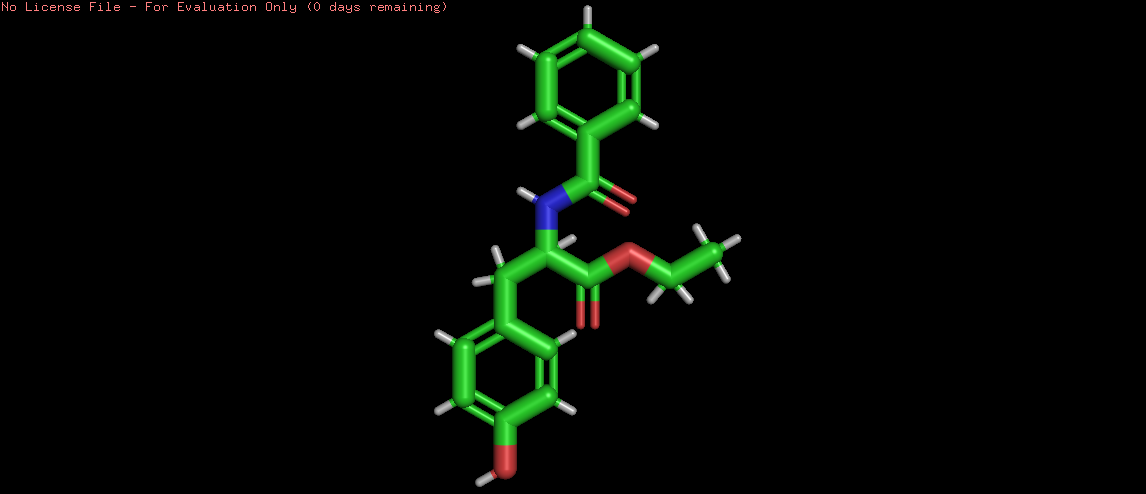 |


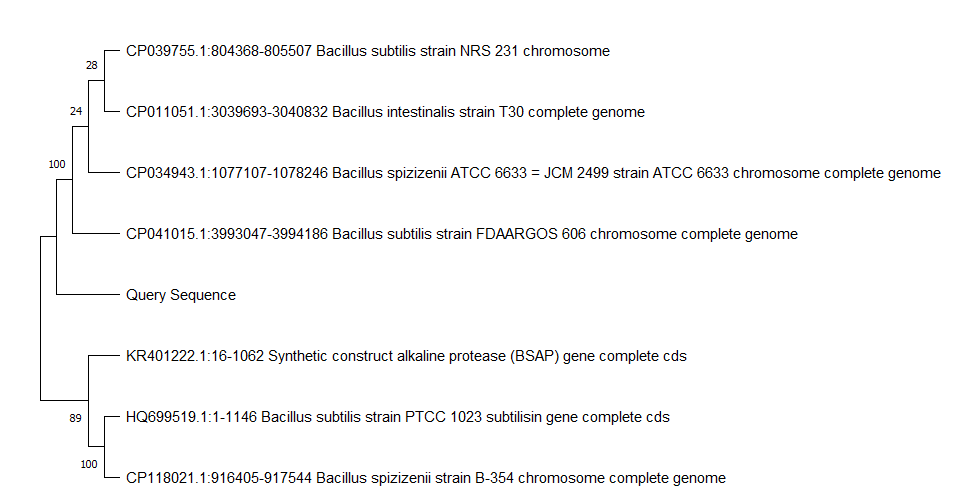


**Fig S1**Phylogenetic relationshipsof subtilisin gene sequence with other genomic sequences employing the neighbor-joining method


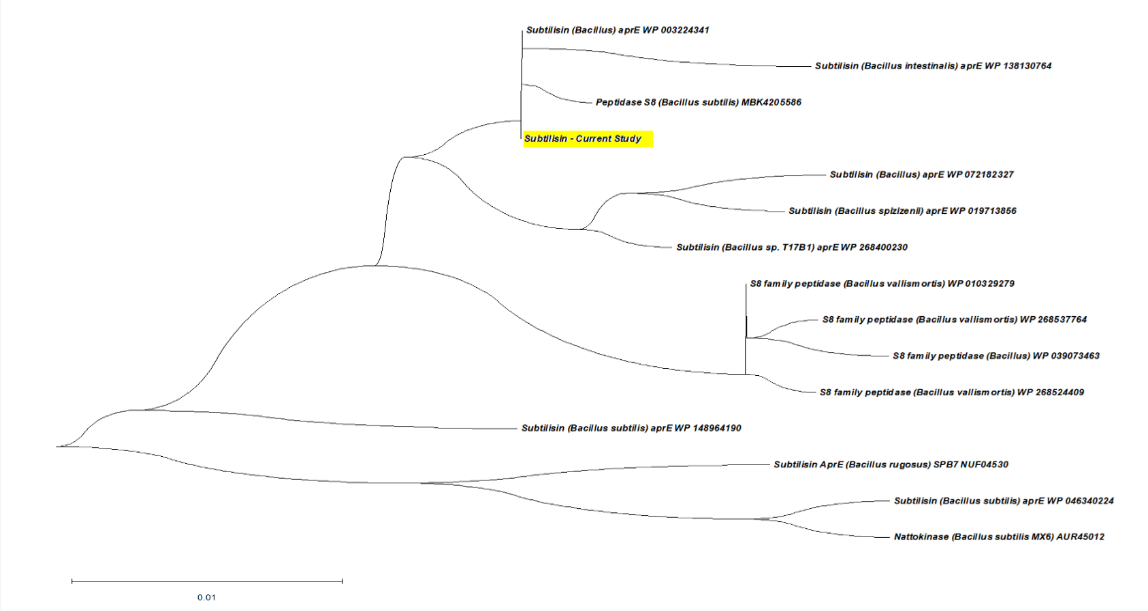


**Fig S2**Evolutionary phylogenetic relationshipsof subtilisin protein sequence with closely related proteins employing the neighbor-joining method


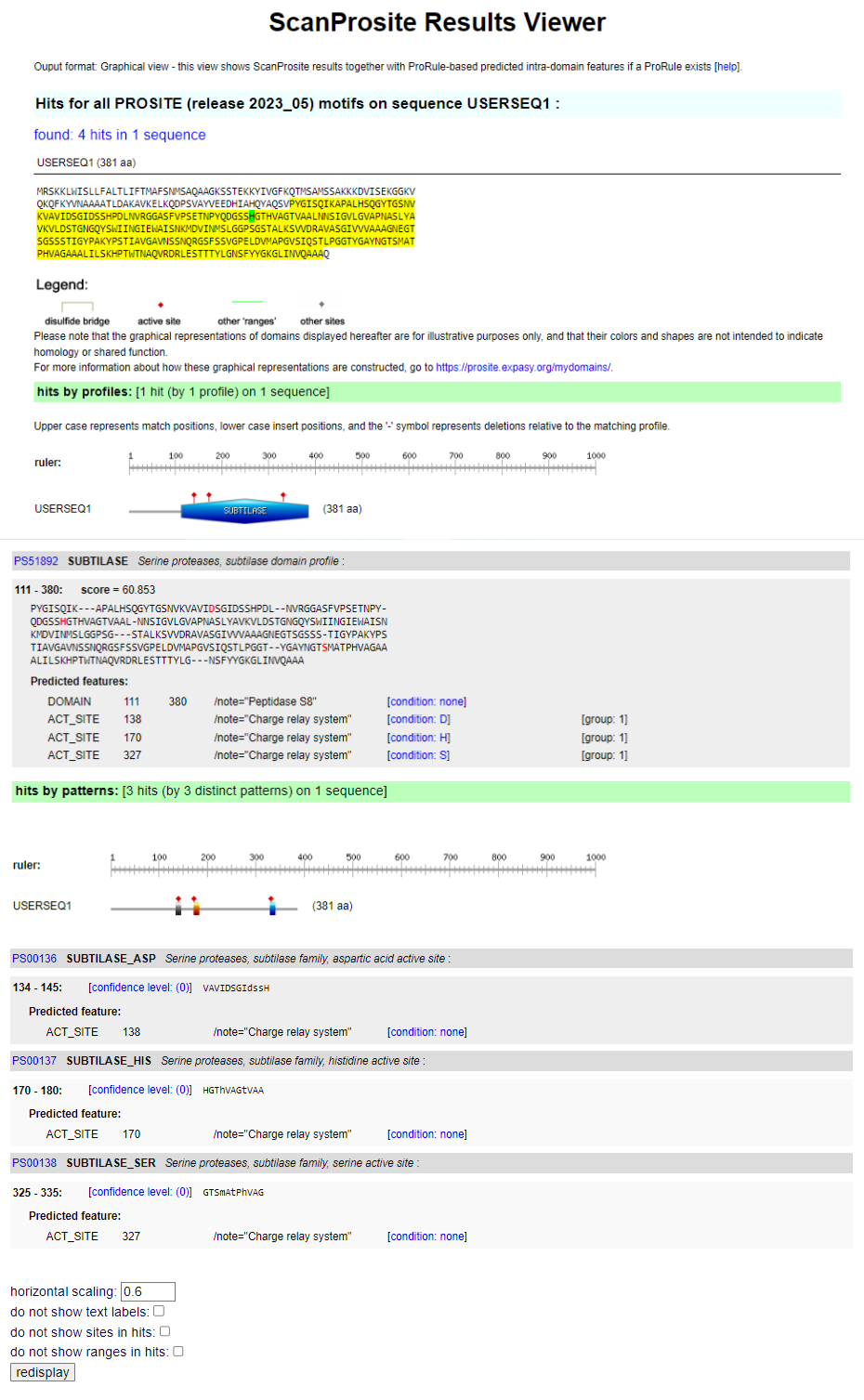


**Fig S3**Prediction of the catalytic domain and active sites using ScanProsite


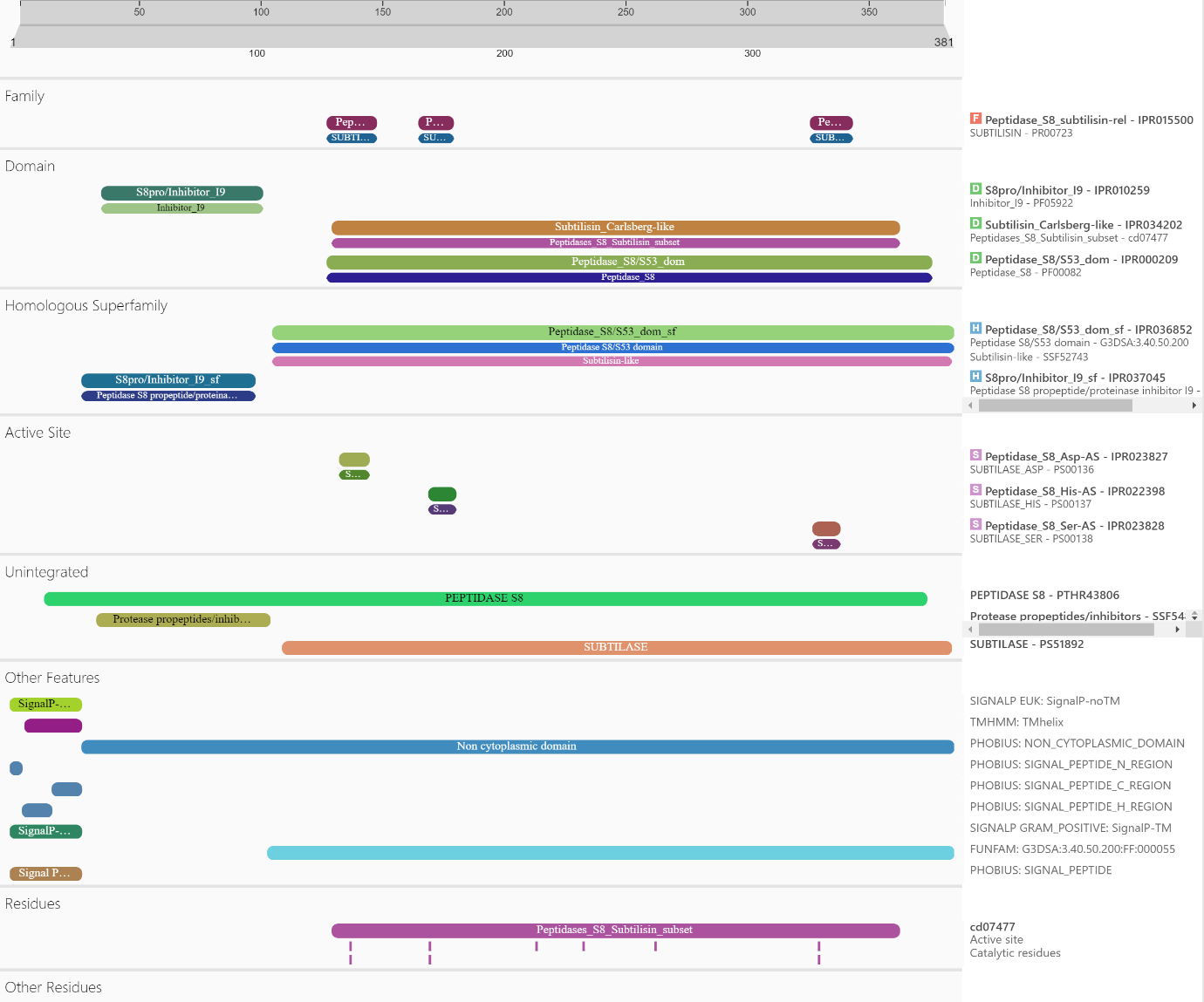


**Fig S4**Conserved domain prediction of subtilisin using InterPro Scan

(A)


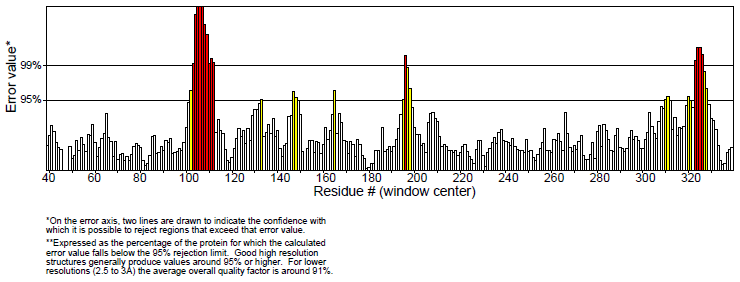


(B)


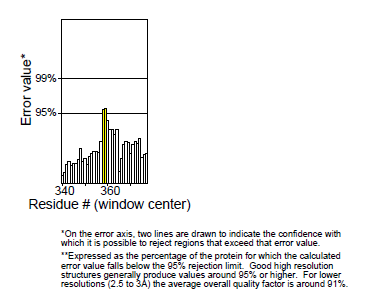


**Fig S5 A and B**Predicted model’s quality factor estimation using ERRAT2


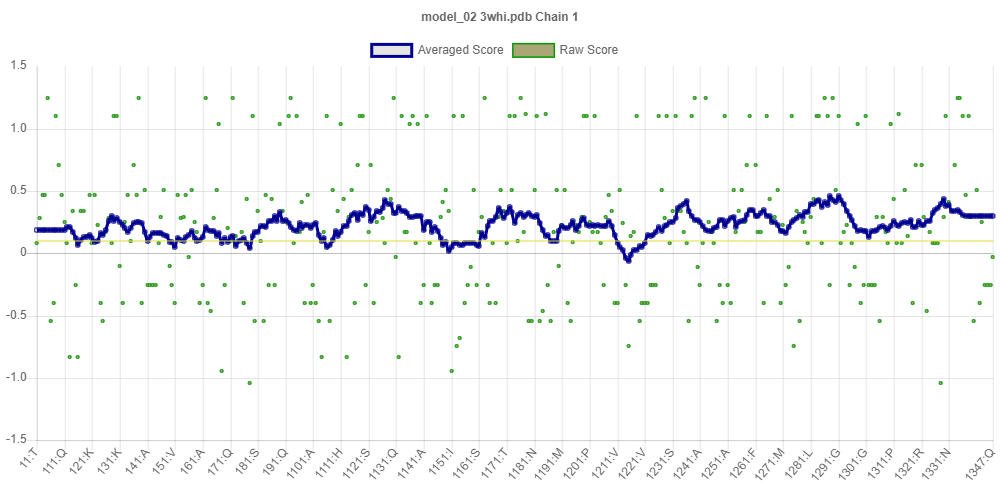


**Fig S6** Evaluation of quality of model using VERIFY3D via SAVES v 6.0


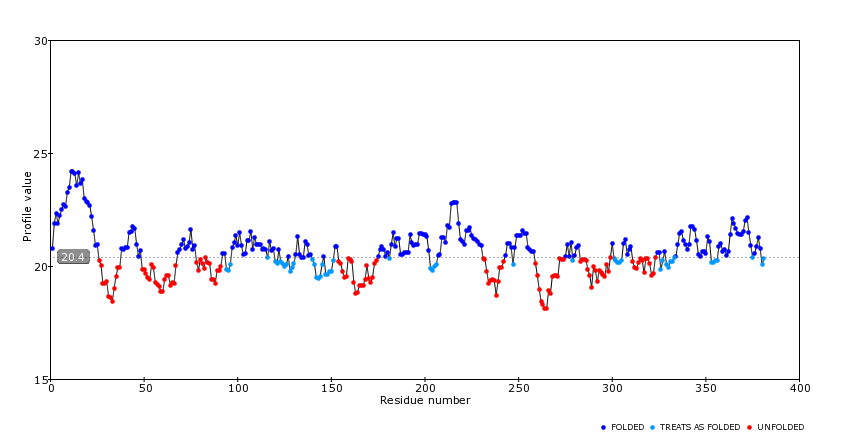


**Fig S7**Prediction of unfolded regions
